# Utilising network pharmacology to explore the underlying mechanism of Wumei Pill in treating pancreatic neoplasms

**DOI:** 10.1186/s12906-019-2580-y

**Published:** 2019-07-04

**Authors:** Yuxiang Wan, Lin Xu, Zeyu Liu, Ming Yang, Xin Jiang, Qiaoli Zhang, Jinchang Huang

**Affiliations:** 1 Beijing University of Chinese Medicine Third Affiliated Hospital, Beijing, 100029 China; 2 School of Traditional Chinese Medicine, Beijing University of Chinese Medicine, Beijing, 100029 China

**Keywords:** Wumei pill, Network pharmacology, Pancreatic neoplasms

## Abstract

**Background:**

Wumei Pill (WMP), a famous herbal formula, has been widely used to treat digestive system diseases in clinical practice in China for centuries. We have found a correlation between the indications of WMP and the typical symptoms of pancreatic neoplasms. However, the pharmacological mechanisms of WMP still remain unknown.

**Methods:**

In the present work, we used a network pharmacological method to predict its underlying complex mechanism of treating pancreatic neoplasms. Firstly, we obtained relative compounds of WMP based on TCMSP database, TCM database@Taiwan and TCMID database and collected potential targets of these compounds by target fishing. Then we built the pancreatic neoplasms target database by CTD, TTD, PharmGKB. Based on the matching results between WMP potential targets and pancreatic neoplasms targets, we built a PPI network to analyze the interactions among these targets and screen the hub targets by topology. Furthermore, DAVID bioinformatics resources were utilized for the enrichment analysis on GO_BP and KEGG.

**Results:**

A total of 80 active ingredients and 77 targets of WMP were picked out. The results of DAVID enrichment analysis indicated that 58 cellular biological processes (FDR < 0.01) and 17 pathways (FDR < 0.01) of WMP mostly participated in the complex treating effects associated with proliferation, apoptosis, inflammatory response and angiogenesis. Moreover, 17 hub nodes of WMP (PTGS2, BCL2, TP53, IL6, MAPK1, EGFR, EGF, CASP3, JUN, MAPK8, MMP9, VEGFA, TNF, MYC, AKT1, FOS and TGFB1) were recognized as potential targets of treatments, implying the underlying mechanisms of WMP acting on pancreatic neoplasms.

**Conclusion:**

WMP could alleviate the symptoms of pancreatic neoplasms through the molecular mechanisms predicted by network pharmacology. This study proposes a strategy to elucidate the mechanisms of Traditional Chinese Medicine (TCM) at the level of network pharmacology.

**Electronic supplementary material:**

The online version of this article (10.1186/s12906-019-2580-y) contains supplementary material, which is available to authorized users.

## Background

Pancreatic Neoplasms are highly malignant neoplasms of digestive system, with an estimation of over 200 thousand fatalities yearly global wide [1]. According to the investigations of the American Cancer Society (ACS), it is suggested that pancreatic neoplasms come in 4th, and appear rising, in the ranking of fatality rate of all malignant tumors, which is predicted to shoot up to 2nd by 2030 [2], with the five-year survival rate of patients under 6% [3, 4]. With the development of molecular biology, the study of systems biology on pancreatic neoplasms has been attracting more and more attention progressively [5, 6].

Traditional Chinese Medicine (TCM) is a branch of traditional oriental medicine, hugely characterized by its systematic and holistic philosophy, which has been practiced by Chinese people for thousands of years due to its satisfying therapeutic effects and minor side effects [7]. Chinese medicine has been playing an important role in the improvement of life quality and extending the lifetime of pancreatic neoplasms patients [8, 9]. Wumei Pill is originated from *Treatise on Cold Damage and Miscellaneous Diseases* (200–210, AD), consisting of 10 herbs: Fructus Mume(WM), Radix Angelicae Sinensis(DG), Rhizoma Radix Asari(XX), Radix Aconiti Lateralis Praeparata(FZ), Cortex Phellodendri(HB), Pericarpium Zanthoxyli(HJ), Ramulus Cinnamomi(GZ), Radix Ginseng(RS), Rhizoma Coptidis(HL) and Rhizoma Zingiberis(GJ). Wumei Pill is broadly applied in clinical treatment of digestive system diseases and have satisfactory effects on upper abdominal distention and pains, diarrhea and loss of appetite [10, 11]. Apart from that, one study showed that Wumei Pill can lower the insulin resistance of HepG2 cells by inhibiting NLRP3 and proinflammatory cytokine [12].

We have found in clinical practice that Wumei Pill can effectively relieve the classic symptoms of pancreatic neoplasms, such as abdominal pain, diarrhea and inappetence [13]. However, the pharmacological mechanisms of Wumei Pill still unknown.

Network Pharmacology is an interactive network based on the concept of “Disease – Gene – Target - Medicine”, standing on a systematic and integrative viewpoint towards the intervention and effect of medication on disease network, in order to reveal the complex mechanism of medicine on human bodies [14]. The systematism of the strategy resonates well with the holistic view of TCM, as well as the mechanism of multi-ingredient, multi-pathway and multi-target synergy in TCM formulas [15–17].

**Fig. 1:**
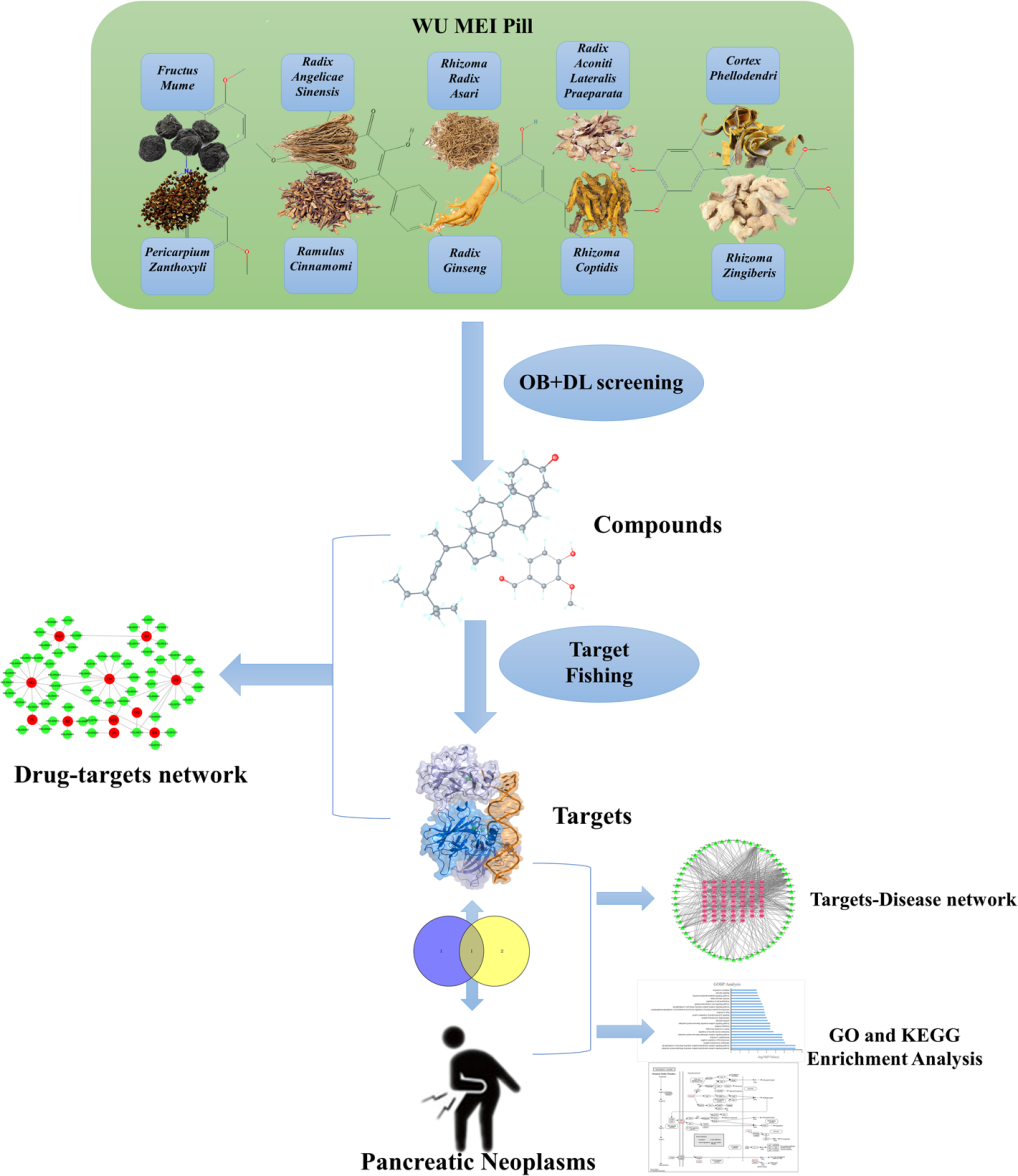
The whole framework based on an integration strategy of network pharmacology

Our study plans to screen out the related ingredients of Wumei Pill via multiple database, and obtain the potential targets by target fishing. Then, we screen out the related targets of pancreatic neoplasms by integration of multi-source database. Based on the matching results between WMP potential targets and pancreatic neoplasms targets, we built a PPI network to analyze the interactions among these targets and screen the hub targets by topology. Furthermore, DAVID bioinformatics resources were utilized for the enrichment analysis on GO_BP and KEGG. Our protocol was shown as Fig. 1.

## Methods

### Chemical ingredients database building

To collect the ingredients of Wumei Pill, we used the Traditional Chinese Medicine System Pharmacology Database [18] (TCMSP™, http://lsp.nwu.edu.cn/tcmsp.php), a unique systemic pharmacology platform designed for Chinese herbal medicinals; The TCM Database@Taiwan [19] (http://tcm.cmu.edu.tw/zh-tw/), which is currently the world’s largest non-commercial TCM database; The Traditional Chinese Medicines Integrated Database [20] (TCMID, http://119.3.41.228:8000/), a comprehensive database to provide information and bridge the gap between Traditional Chinese Medicine and modern science. Nine hundred fifty-three herbal ingredients were found in this process.

### Active ingredients screening

#### OB prediction

Oral Bioavailability (OB) is the percentage of an oral administered drug that reaches systemic circulation, which is one of the most generally used pharmacokinetic properties in drug screening. A robust in-house system OBioavail1.1 integrates the metabolism (P450 3A4) and transport (P-glycoprotein) information to calculate the OB value [21]. In this process, the OB threshold was set as 30% and those ingredients with OB≧30% were selected as the active ingredients for next step.

#### DL evaluation

Drug-likeness (DL) is a molecular parameter that measures absorption, distribution, metabolism and excretion of drug molecules influenced by its pharmacokinetics, and it is also a qualitative concept used in drug design for an estimate on how “drug-like” a prospective compound is, which helps to optimize pharmacokinetic and pharmaceutical properties, such as solubility and chemical stability. In this process, Tanimoto Similarity (TS) was used to select ingredients considered chemically suitable for drugs [22]. The TS index is shown as follows.

1 $$ \mathrm{T}\left(\mathrm{A},\mathrm{B}\right)=\frac{A\cdot B}{\parallel A{\parallel}^2+\parallel B{\parallel}^2-A\cdot B} $$

In the eq. (1), A represents the molecular descriptors of herbal ingredients, and B displays the average molecular properties of all ingredients in Drugbank. The DL level of the compounds is 0.18, which is used as a selection criterion for the “drug-like” compounds in the traditional Chinese herbs [23]. In this study, those ingredients with DL≧0.18 were preserved. Finally, herbal ingredients were chosen as the candidate ingredients for further analysis when they met both these two criteria.

#### Caco-2 permeability

The absorption of orally administered drugs is mainly in the small intestine where the surface absorptivity is greatly increased by the presence of villi and microvilli [24]. Thus, the in silico Caco-2 permeability model which was constructed by 100 drug molecules with satisfactory statistical results (R^2^>0.8) was employed to select compounds that are more likely to possess good permeability [25]. Eventually, considering that compounds with Caco-2 value less than 0 is not permeable, the threshold of Caco-2 permeability is set at 0.

#### Target fishing

The active ingredients of drugs exert related biological function via targets. Our study located related targets by target fishing based on aforementioned included active ingredients. On the retrieval on Pubchem database of the micromolecular structure information of the active ingredients of Wumei Pill, we fished targets according to the chemical similarity, screening via online tools such as Similarity ensemble approach (SEA, http://sea.bkslab.org/) [26], Swiss Target Prediction webserver (http://www.swisstargetprediction.ch/index.php) [27] and SuperPred webserver (http://prediction.charite.de/) [28].

#### Disease targets database building

Related targets of pancreatic neoplasms were collected by the integration of multi-source databases. Databases used in our study were as follows: Comparative Toxicogenomics Database (CTD, http://ctdbase.org/) [29], Therapeutic Target Database (TTD, https://db.idrblab.org/ttd/) [30] and PharmGKB (https://www.pharmgkb.org/) [31]. We searched these databases with the keyword “Pancreatic cancer”. Two hundred forty-five targets were found in this process.

At last, we matched the prediction of the targets of Wumei Pill active ingredients and the retrieval of the related targets of pancreatic neoplasms, then chose the overlapping targets as the related targets of Wumei Pill in treating pancreatic neoplasms. The targets were then processed by String [32] (https://string-db.org/) to draw the data of protein-protein interaction (PPI).

### Network construction

#### Network construction method

In this process, Network construction was performed as follows: 1. Compound-target network (C-T network). 2. WMP target-Pancreatic cancer target interactional network (T-T network). 3. Target-pathway network (T-P network). The pathway information of targets was selected from the result of KEGG pathway enrichment. All visualized network graphs were established by Cytoscape3.6.0 (http://www.cytoscape.org/), an open-source software platform for visualizing complex networks and integrating these with any type of attribute data [33]. However, the interaction relationships of the nodes in these networks, such as the action type e.g. activation, inhibition, binding, catalysis, the action effect e.g. positive, negative, unspecified, and so on are still not clear, which is the limitation of this study.

#### Network topological feature set definition

For each node in the interaction network, we selected three parameters to assess its topological features: 1. “Degree” is defined as the number of links to one node and reflects how often one node interacts with other nodes [34]. 2. “Betweenness Centrality” measures the extent to which a node lies on paths between other nodes. Nodes with high betweenness may have considerable influence within a network by virtue of their control over information passing between others [35]. 3. “Closeness Centrality” measures the mean distance from a node to other nodes. A geodesic path is a shortest path through a network between two nodes [36]. The three parameters play an crucial role in the network, and the level of the three parameters represent the topological importance of the nodes in the network, with more important nodes outputting higher values in the network. The threshold values of the hub nodes in the network analysis is the corresponding median values of each parameters.

#### Enrichment analysis

We used the Database for Annotation, Visualization and Integrated Discovery [37] (DAVID, https://david.ncifcrf.gov/, v6.8) for gene ontology (GO) enrichment analysis, and also accomplished pathway enrichment analysis using Kyoto Encyclopedia of Genes and Genomes [38] (KEGG, http://www.kegg.jp/) data obtained from DAVID.

## Results

### Active compounds of Wumei pill

**Fig. 2:**
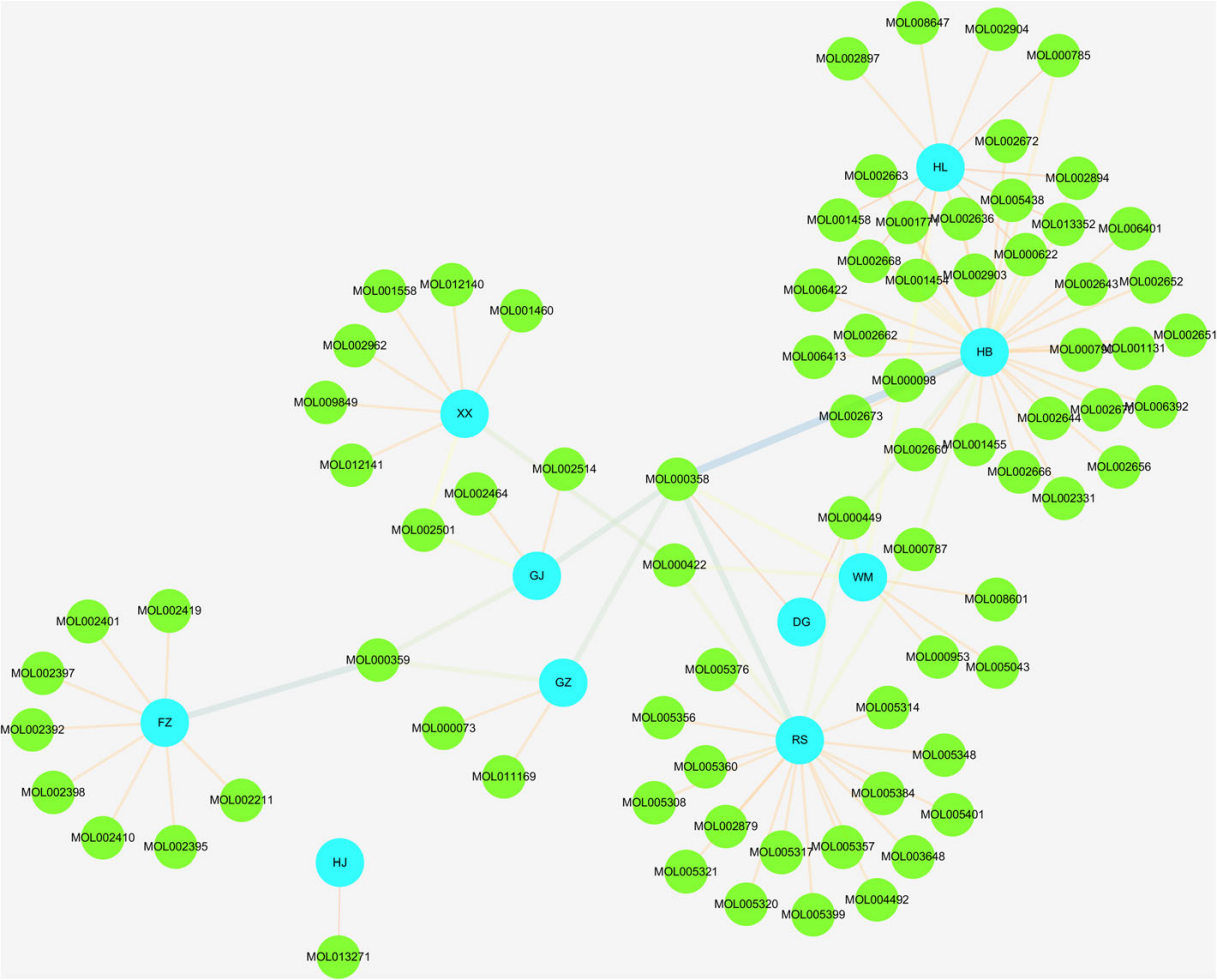
Herbs-Compounds network. The blue nodes represent herbs in Wumei Pill, and the green nodes represent active compounds. The edges represent the relationship between them, and the length, width and color of the edges is proportional to the edge betweenness

Retrieved from three following databases: TCMSP, TCM Database@Taiwan and TCMID, there were 953 related components of the whole formula in total, among which there were 40 (4.2%) of WM, 192 (20.1%) of XX, 149 (15.6%) of GJ, 48 (5%) of HL, 65 (6.8%) of FZ, 125 (13.1%) of DG, 140 (14.7%) of HB, 221 (23.2%) of GZ, 190 (19.9%) of RS and 30 (3.1%) of HJ. According to the ADME thresholds of OB≧30%, DL≧0.18 and Caco-2>0, 80 active ingredients were selected out (Additional file 1: Table S1) and the Herbs-Compounds network were built as Fig. 2. After the construction of the Herbs-Compounds network and analysis of the 80 active ingredients, in descending order of edge betweenness, the top four ingredients were beta-sitosterol (MOL000358, OB = 36.91, DL = 0.75, Caco-2 = 1.32, found in WM, GJ, DG, GZ, RS, HB), sitosterol (MOL000359, OB = 36.91, DL = 0.75, Caco-2 = 1.32, found in GJ, FZ, GZ), kaempferol (MOL000422, OB = 41.88, DL = 0.24, Caco-2 = 0.26, found in WM, XX, RS) and Stigmasterol(MOL000449, OB = 43.83, DL = 0.76, Caco-2 = 1.44, found in WM, RS, DG, HB).

### Target prediction and analysis

**Fig. 3:**
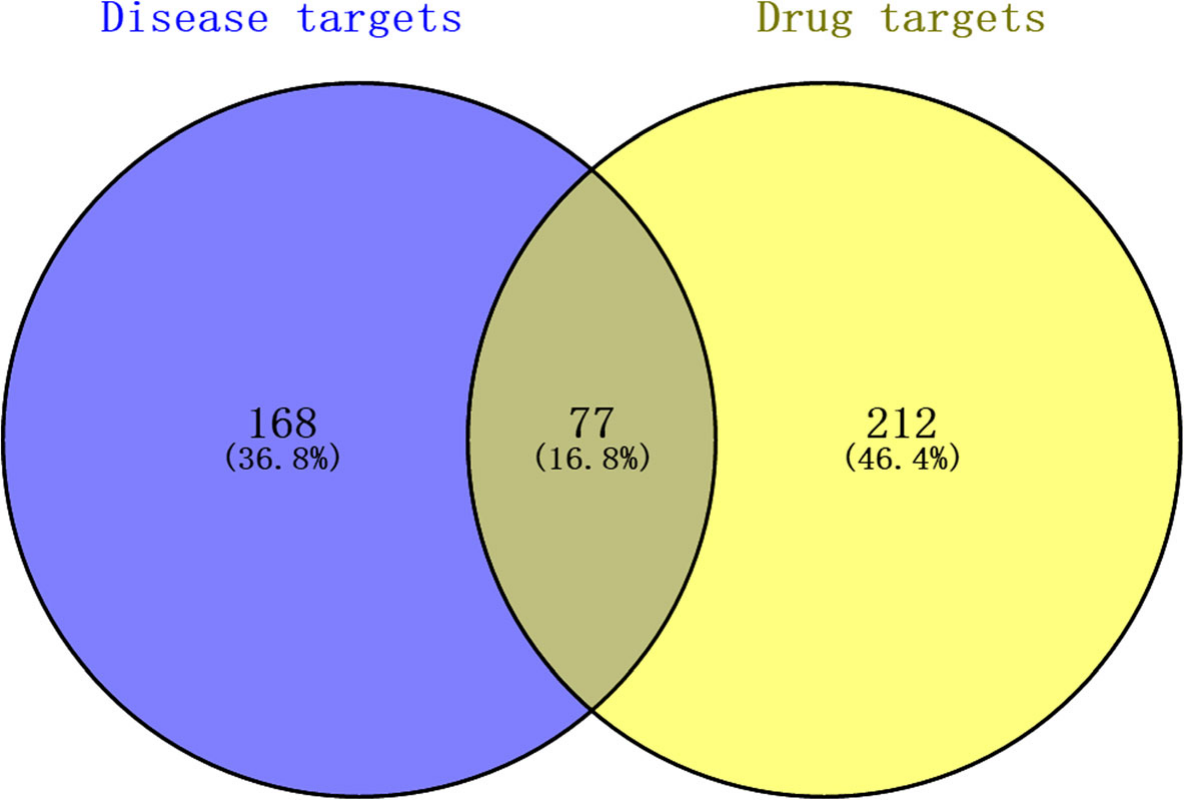
The venn diagram of the targets both in pancreatic cancer targets and Wumei pill targets

In this process we conducted target fishing on the 80 active compounds based on chemical similarity, obtaining 289 related targets, among which there were 189 in WM, 108 in XX, 94 in RS, 52 in DG, 68 in FZ, 44 in GJ, 43 in GZ, 236 in HB, 3 in HJ and 189 in HL. Via the method of integration of multi-source databases, the target data of pancreatic neoplasms from databases of CTD, TTD and PharmGKB was integrated. Then we selected the top 200 genes related to pancreatic neoplasms based on Inference Score from CTD database as major targets of pancreatic neoplasms, with a total of 245 related targets. Seventy-seven matching targets of the related targets of Wumei Pill and pancreatic neoplasms were then collected as the related targets of anti-cancer effect of Wumei Pill on pancreatic neoplasms (Fig. 3.).

**Fig. 4:**
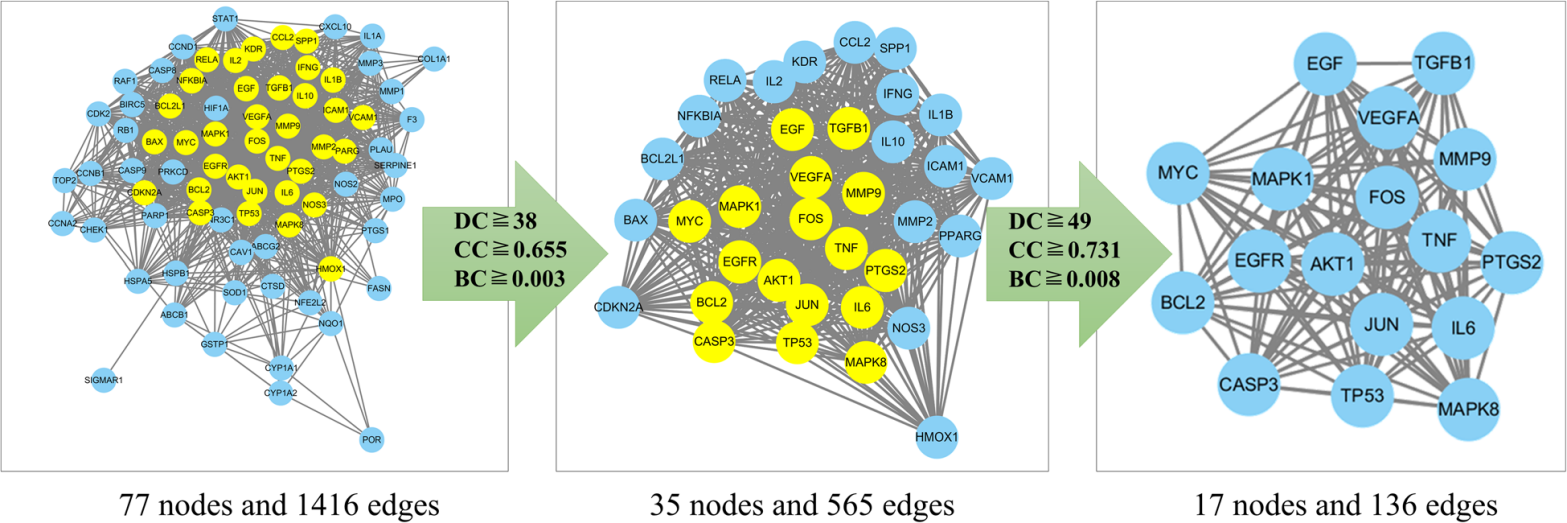
The process of topological screening for the PPI network

**Fig. 5:**
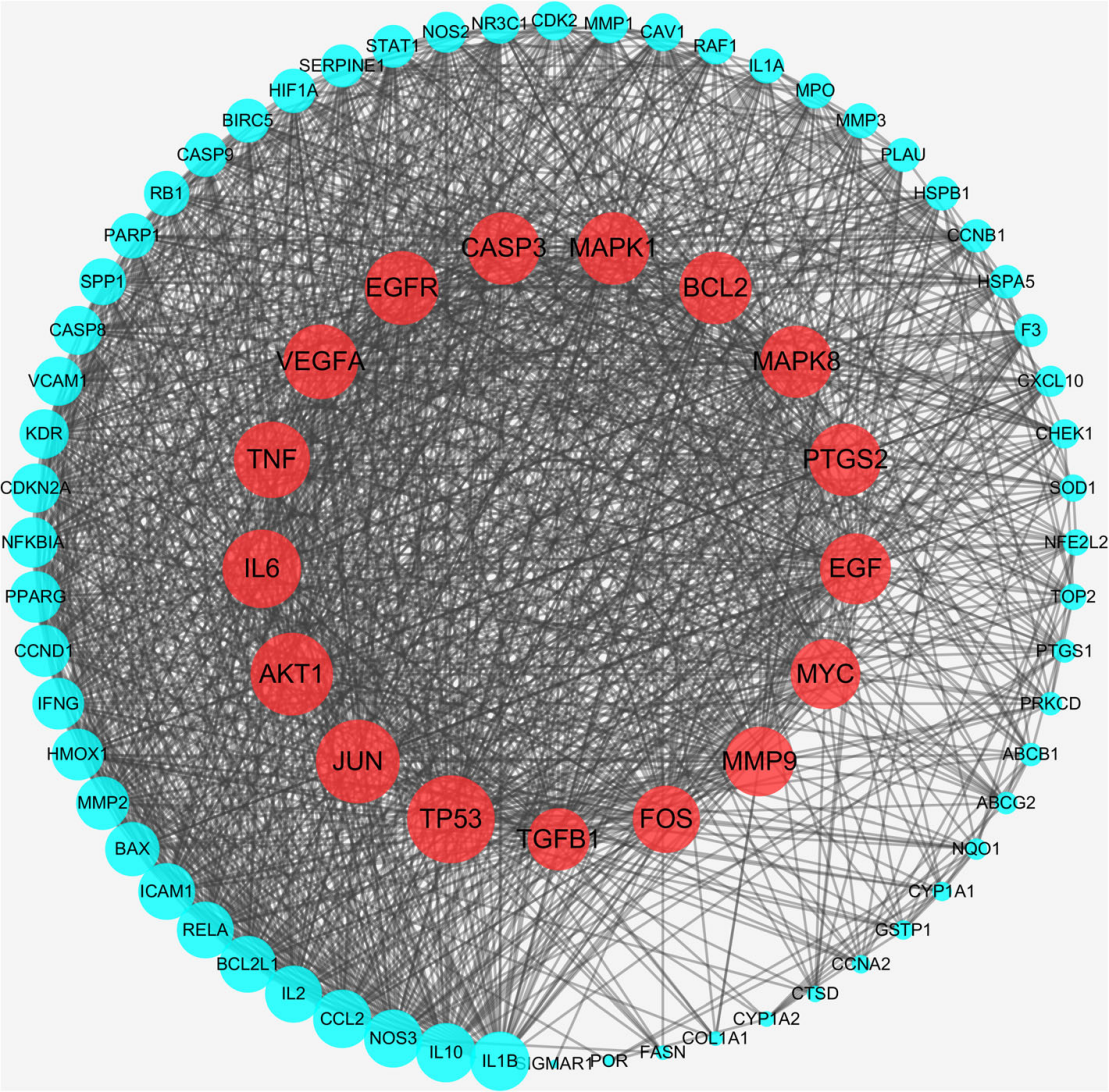
The PPI network of 77 nodes. The red nodes represent the big hub nodes, the blue nodes represent the other nodes. The node size is proportional to the target degree in the network

In the database of String, Protein-Protein Interaction (PPI) network of those 77 targets were later established. There were 77 nodes and 1416 edges in total. The topological feature analysis of the aforementioned PPI afterwards was based on three major parameters of “degree”, “betweenness” and “closeness”, selected targets above median values as key targets and constructed the big hub nodes of the anti-cancer effect of Wumei Pill on pancreatic neoplasms. The threshold values of the first screening were degree≧38, closeness≧0.655172 and betweenness≧0.003193, and the results settled at 35 hub nodes and 565 edges. The 35 key targets were then further screened with the second screening threshold values as degree≧49, closeness≧0.73076923, betweenness≧0.00899538. The second screening ended up with 17 big hub nodes and 136 edges (Fig. 4.), which included, in specification, PTGS2, BCL2, TP53, IL6, MAPK1, EGFR, EGF, CASP3, JUN, MAPK8, MMP9, VEGFA, TNF, MYC, AKT1, FOS and TGFB1 (Table 1.). When the 17 big hub nodes and other 60 nodes are sorted in descending order and viewed in network, TP53 (degree = 71), JUN (degree = 68), AKT1 (degree = 67), IL6 (degree = 64), TNF (degree = 62), VEGFA (degree = 61) and EGFR (degree = 60) are were the key targets in this network (Fig. 5.).

**Table 1:** Information on 17 hub targets

Uniprot ID	Gene symbol	Protein name	Degree
P04637	TP53	Cellular tumor antigen p53	71
P05412	JUN	Transcription factor AP-1	68
P31749	AKT1	RAC-alpha serine/threonine-protein kinase	67
P05231	IL6	Interleukin-6	64
P01375	TNF	Tumor necrosis factor	62
P15692	VEGFA	Vascular endothelial growth factor A	61
P00533	EGFR	Epidermal growth factor receptor	60
P42574	CASP3	Caspase 3	59
P28482	MAPK1	Mitogen-activated protein kinase 1	59
P10415	BCL2	Apoptosis regulator Bcl-2	59
P45983	MAPK8	Mitogen-activated protein kinase 8	59
P35354	PTGS2	Prostaglandin G/H synthase 2	59
P01133	EGF	Pro-epidermal growth factor	58
P01106	MYC	Myc proto-oncogene protein	57
P14780	MMP9	Matrix metalloproteinase-9	57
P01100	FOS	Proto-oncogene c-Fos	55
P01137	TGFB1	Transforming growth factor beta-1	51

**Fig. 6:**
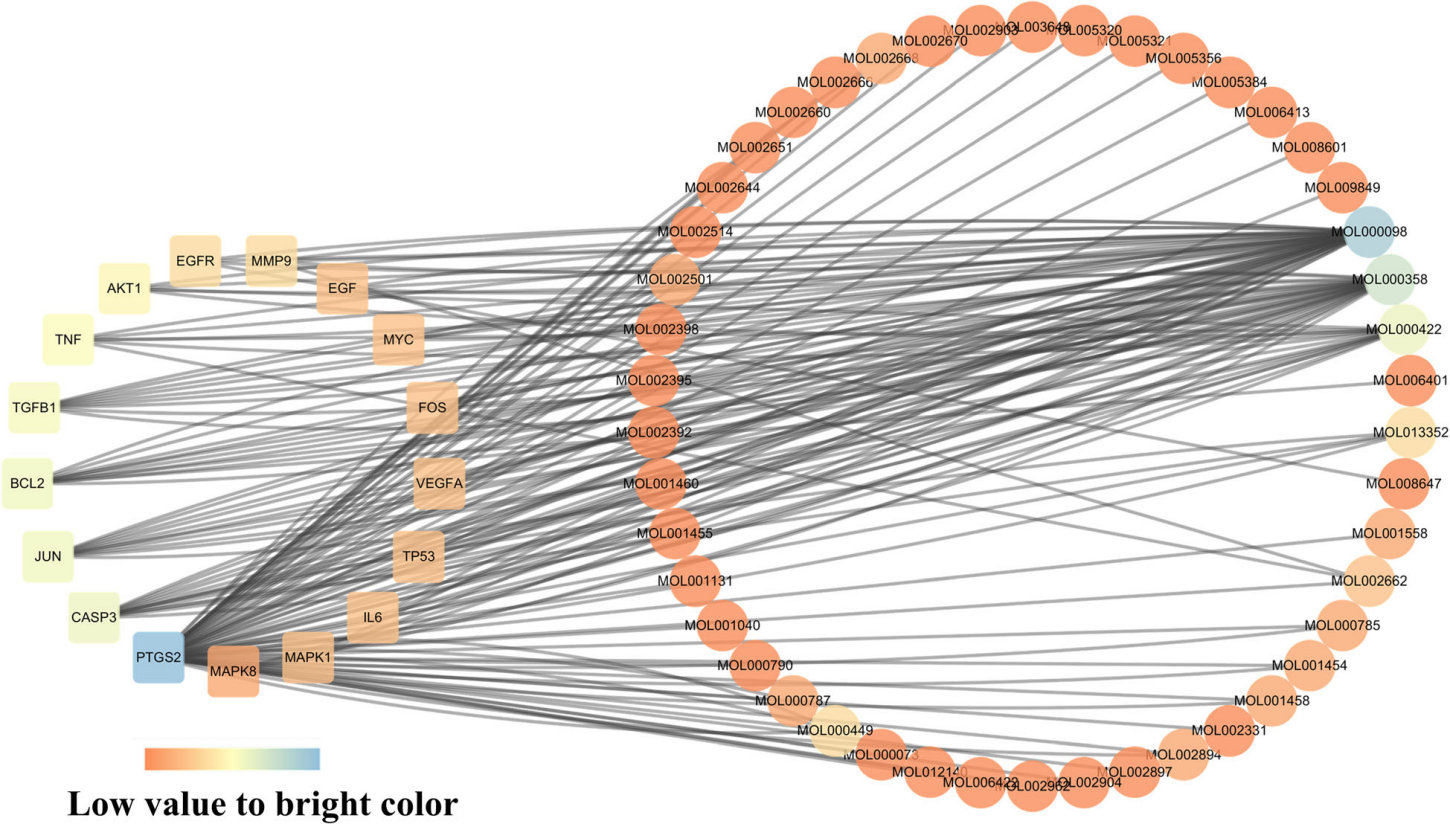
Big hub nodes-Compounds Network. The square nodes represent the big hub nodes, the round nodes represent the compounds. The node color changes from orange to blue reflect the degree value changes from low to high in the network

Based on 17 key targets, we further constructed the Big hub nodes—Compounds Network (Fig. 6.) according to the related active components. In this network, there were 46 active components, including quercetin (MOL000098), which held relevancy to 16 key targets, kaempferol (MOL000422), which was related to 7 key targets, beta-sitosterol (MOL000358), related to 5 key targets, rutaecarpine (MOL002662)with connection to 3 key targets and Obacunone (MOL013352) linked to 2 key targets.

### GO biological process and KEGG pathway enrichment analysis

The enrichment analysis performed with DAVID v6.8 on 77 targets, screening threshold being FDR<0.01, had retrieved 58 GO items; and we have selected 17 related KEGG pathways for analysis.

### GO biological process enrichment analysis

**Fig. 7:**
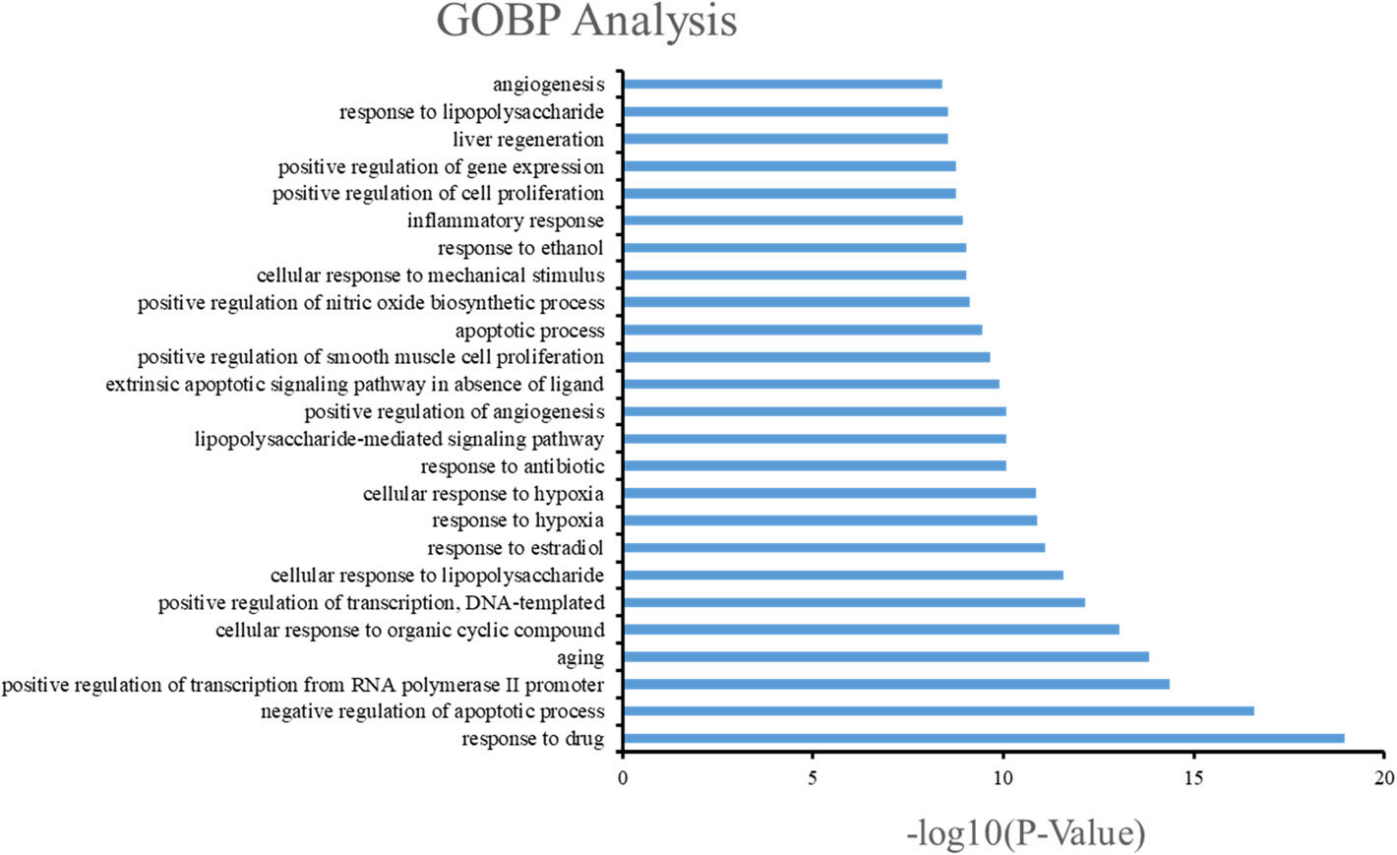
The GOBP enrichment analysis of 77 nodes

After sorting 58 items based on FDR values, the top 25 biological processes (Fig. 7.) were mainly involved in cell apoptosis, proliferation, inflammatory response and angiogenesis. In the aspect of apoptosis, we mainly had: negative regulation of apoptotic process (GO:0043066), apoptotic process (GO:0006915), extrinsic apoptotic signaling pathway in absence of ligand (GO:0097192); regarding the regulation of proliferation, we found: positive regulation of transcription from RNA polymeraseIIpromoter (GO:0045944), positive regulation of DNA-templated transcription (GO:0045893), positive regulation of smooth muscle cell proliferation (GO:0048661), positive regulation of cell proliferation (GO:0008284) and positive regulation of gene expression (GO:0010628); in the aspect of regulation of inflammatory response, there were: inflammatory response (GO:0006954), cellular response to lipopolysaccharide (GO:0071222), response to antibiotic (GO:0046677) and lipopolysaccharide-mediated signaling pathway (GO:0031663); and in the aspect of angiogenesis, we had: response to hypoxia (GO:0001666), positive regulation of angiogenesis (GO:0045766) and angiogenesis (GO:0001525). Based on those four main aspects above, it is possible that the anti-cancer effect of Wumei Pill on pancreatic neoplasms results from a complex multi-pathway synergetic effect.

### KEGG pathway enrichment analysis

**Fig. 8:**
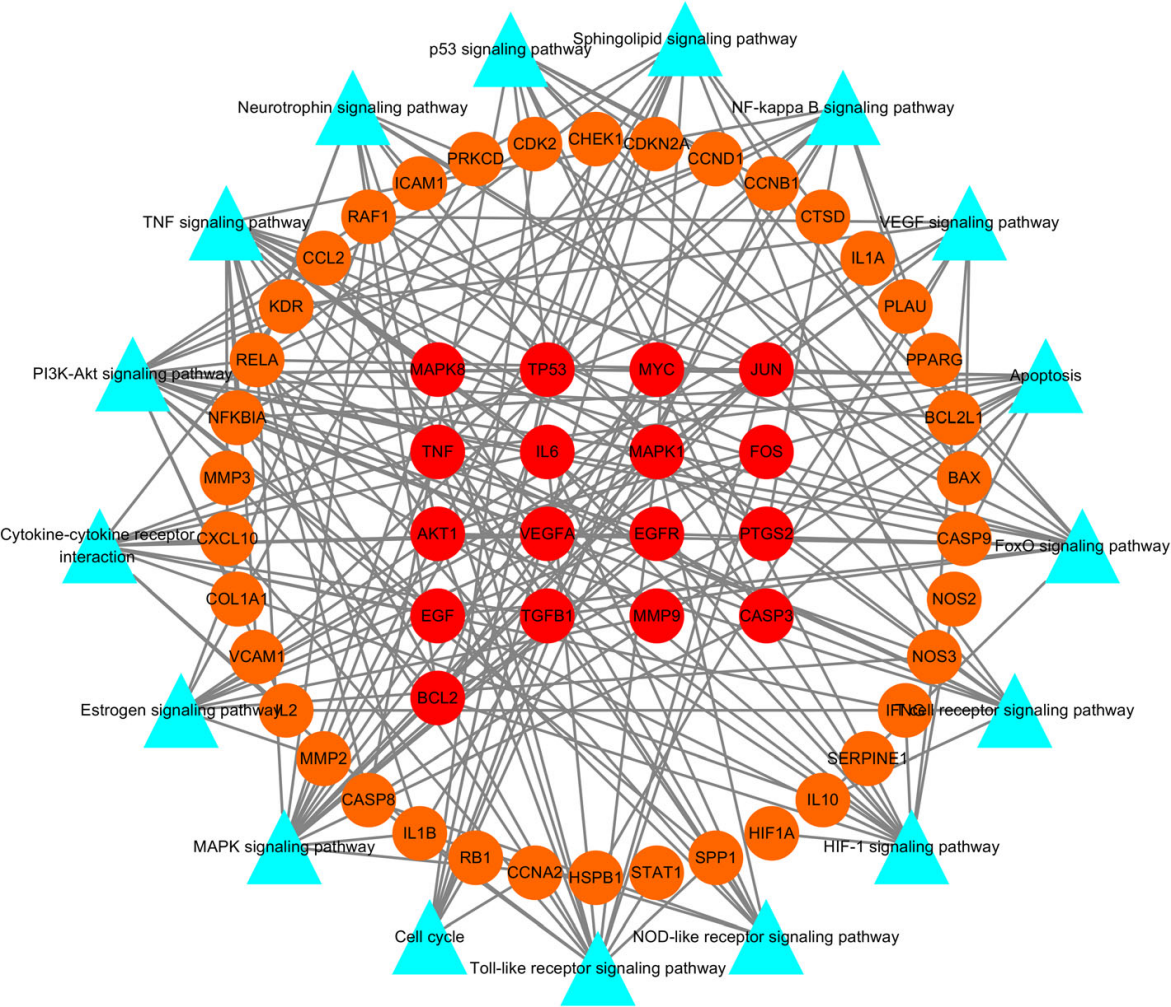
Target-Pathway Network. The red round nodes represent the big hub nodes, the orange round nodes represent the other nodes. The blue triangles represent the related pathways

To further reveal the potential mechanism of the anti-cancer effect of Wumei Pill on pancreatic neoplasms, we conducted KEGG pathway enrichment analysis on 77 targets and screened out 17 pathways based on the threshold of FDR<0.01 and cancer-related signaling pathways, for instance, TNF signaling pathway (hsa04668), HIF-1 signaling pathway (hsa04066), Apoptosis (hsa04210), p53 signaling pathway (hsa04115), PI3K-Akt signaling pathway (hsa04151), VEGF signaling pathway (hsa04370), NF-kappa B signaling pathway (hsa04064), etc.(Table 2.). Meanwhile, we established the Target-Pathway Network based on the targets of Wumei Pills on each pathway (Fig. 8.).

**Table 2:** Information on 17 pathways

Term ID	Pathway name	Count	FDR
hsa04668	TNF signaling pathway	19	1.62E-14
hsa04620	Toll-like receptor signaling pathway	14	6.07E-08
hsa04066	HIF-1 signaling pathway	13	3.68E-07
hsa04210	Apoptosis	11	7.81E-07
hsa04115	p53 signaling pathway	11	1.73E-06
hsa04151	PI3K-Akt signaling pathway	20	1.87E-06
hsa04010	MAPK signaling pathway	17	7.26E-06
hsa04621	NOD-like receptor signaling pathway	9	1.05E-04
hsa04660	T cell receptor signaling pathway	11	1.22E-04
hsa04068	FoxO signaling pathway	12	1.51E-04
hsa04370	VEGF signaling pathway	9	2.42E-04
hsa04064	NF-kappa B signaling pathway	10	3.22E-04
hsa04071	Sphingolipid signaling pathway	11	5.19E-04
hsa04722	Neurotrophin signaling pathway	11	5.19E-04
hsa04060	Cytokine-cytokine receptor interaction	14	7.88E-04
hsa04915	Estrogen signaling pathway	10	9.83E-04
hsa04110	Cell cycle	10	0.006527

## Discussion

We found that the Wumei Pill could moderate symptoms of pancreatic cancer patients apparently and we thought that the effects may have some relationship with the potential anti-pancreatic cancer function. Therefore, we try to explore the potential mechanism by the network pharmacology approach.

According the Herbs-Compounds network, we found the four hub compounds: beta-sitosterol, sitosterol, kaempferol and Stigmasterol. Among of them, beta-sitosterol has shown potent anti-pancreatic cancer activity by effectively inhibited the growth of tumor cell lines by inhibiting proliferation [39]. Kaempferol is a flavonoid, has antioxidant and anti-pancreatic cancer effect by inhibiting pancreatic cancer cell growth via PI3K/AKT pathway [40]. To conclude, these active ingredients are the material basis of the potential anti-cancer effect of Wumei Pill on pancreatic neoplasms.

**Fig. 9:**
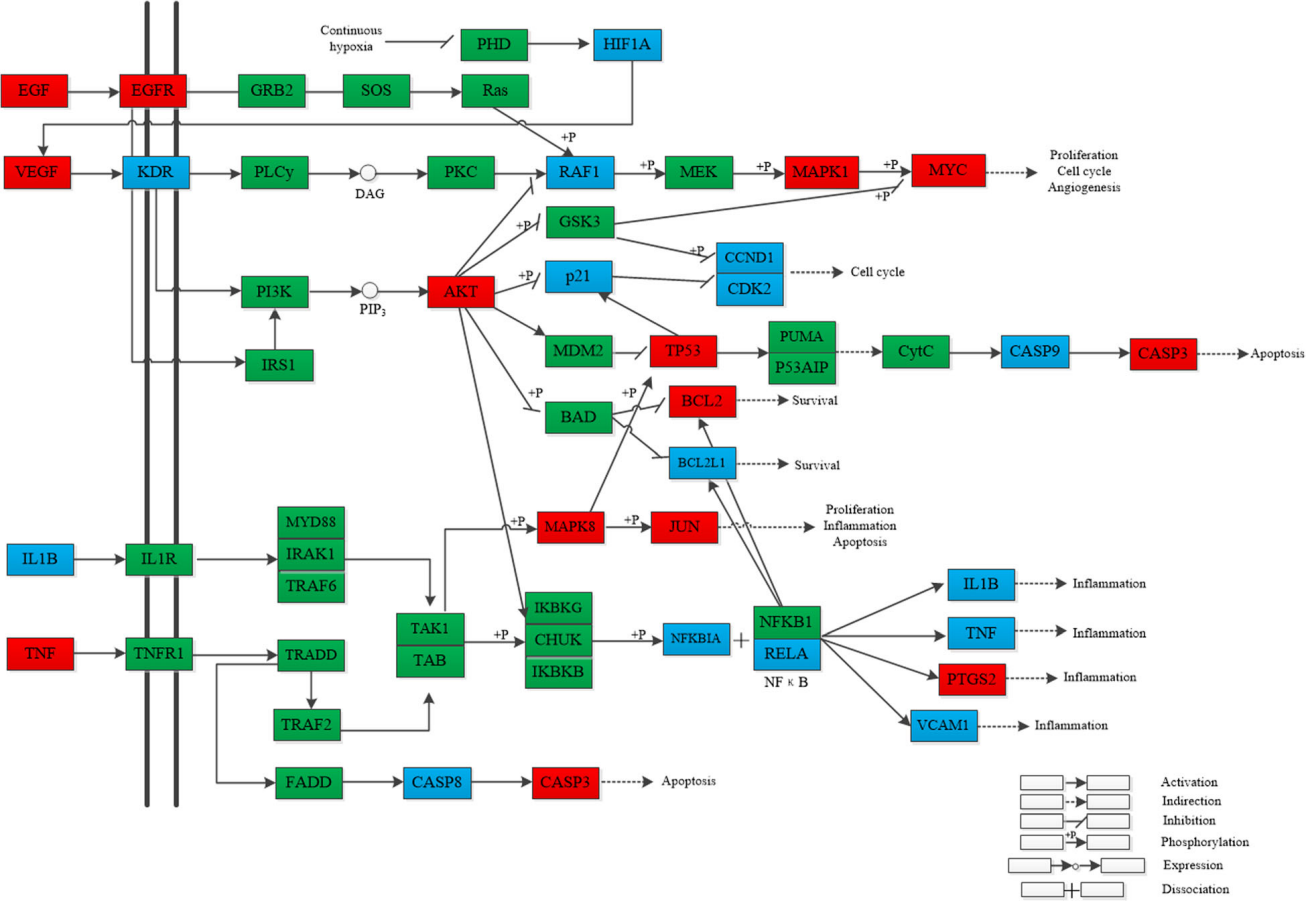
The anti-pancreatic cancer pathway of Wumei Pill. The red nodes represent the big hub nodes, the blue nodes represent the other nodes, and the green nodes represent the targets in the pathway

We came at the speculation with analysis and observation of Target-Pathway network, combined with the GO analysis results, that the potential mechanism of treating pancreatic neoplasms of Wumei Pill is probably connected to its participance in biological processes of proliferation, apoptosis, inflammatory response and angiogenesis (Fig. 9.).

### Cell proliferation

Abnormal proliferation of cells is one of the key characteristics of tumors. EGF is the earliest identified growth factor and plays an important role in the regulation of the growth, proliferation and differentiation of cells [41]. The combination of EGF and EGFR causes the dimerization of EGFR, further activates the PI3K-AKT pathway and eventually the proliferation of cells [42, 43]. TP53 is an important tumor suppressor gene, its regulative effect on cell cycle fulfilled by target gene p21 [44]. Furthermore, MAPK is capable of activating other protein kinases and transcription factors, which further promotes the growth and proliferation of cells.

Wumei Pill’ s regulative effect on cell proliferation is possibly achieved by the inhibitory effect of quercetin, which is shared by Mume Fructus, Coptidis Rhizoma and Phellodendri Chinensis Cortex, on the expression level of EGF and EGFR [45], which further regulates the PI3K-Akt signaling pathway. For instance, quercetin and kaempferol inhibits the expression of AKT1 to effect downstream targets, thus realize the inhibition of cell proliferation. Also, quercetin can positively regulate DNA repair by acting on TP53 and affecting cell cycle. Besides, quercetin is capable of intervening RAS-RAF-MAPK pathway via acting on RAF1 and MAPK1, further regulating cell proliferation.

### Cell apoptosis

In the growth and development of tumors, the inhibition of cell apoptosis plays a vital role alongside excessive proliferation of cells [46]. The expression and regulation of the BCL-2 family is one of the key factors in cell apoptosis, among which BCL-2, BCL-xL and BCL-w are classified as inhibitor of apoptosis proteins, while BAX, BAK and BAD are categorized as pro-apoptotic proteins [47]. These proteins are all regulators of cell apoptosis in PI3K-AKT pathway and P53 pathway [48, 49].

In Wumei Pill, components that acts on BCL-2 family include: quercetin, beta-sitosterol, kaempferol and melianone. Among them, quercetin, beta-sitosterol and kaempferol can not only act on the pro-apoptotic protein BAX, but also the inhibitor of apoptosis proteins BCL-2 and BCL-xL, which may be a two-way regulation of cell apoptosis. Besides, the direct or indirect regulation of quercetin, beta-sitosterol and Obacunone on CASP9 and CASP3 in CASP cascade reaction is possibly an important component of the regulation of cell apoptosis from Wumei Pill [50].

### Inflammatory response

The stimuli of inflammatory response can cause tumor to release multiple factors promoting the growth of itself, which forms an inflammatory microenvironment [51]. TNF is one of the important pro-inflammatory factors, which can not only participate in cell apoptosis, but also mediate the survival and proliferation signals of cells [52]. NF-κB is a key factor in inflammatory response, which can not only enter cell nucleuses, accelerating the further expression of inflammatory factors, such as IL1B, TNFA and PTGS2, but also block apoptosis by activating inhibitor of apoptosis proteins BCL-2 and BCL-XL, and participate in angiogenesis and tumor spreading by regulating VEGF and IL-8 [53].

It is possible that components such as quercetin, kaempferol and rutaecarpine in Wumei Pill affect the entire inflammatory response by inhibiting the expression of TNF in the upstream. There’s another possibility that they participate in NF-kappa B signaling pathway and down-regulate NFKBIA, inhibiting the release of NF-kappa B directly or indirectly, so as to inhibit the expression of downstream inflammatory factors [54]. Studies showed that cancer pain is strongly linked to the participance of certain inflammatory factors [55], and judging from which, the relief from Wumei Pill of pain from pancreatic neoplasms may be somewhat related to its anti-inflammatory effect.

### Angiogenesis

The growth and metastasis of tumor is dependent on angiogenesis [56]. Hypoxia, as an essential characteristic of tumor microenvironment (TME), can stimulate the transcription of hypoxia-inducible factor HIF gene, consequently activate the expression of proangiogenic factor VEGF [57]. VEGF is an important proangiogenic factor, when combined with VEGFR, can activate a series of downstream signaling molecules, such as PLC-γ, PKC, PI3K, MAPK, etc., and eventually relay the signal into cell nucleus and realize the biological effect of VEGF by the expression of certain genes [58].

It is possible that the formula of Wumei Pill can exert the anti-cancer effect by acting on HIF1A and inhibiting the expression level of VEGFA and its binding capacity with KDR, consequently regulating negatively the activation of downstream signaling molecules, eventually inhibiting the angiogenesis of tumors.

## Conclusion

Via the method of Network Pharmacology, our study has predicted the targets of the ingredients of the Wumei pill and explored the underlying mechanism of the potential anti-pancreatic cancer effects which is likely to focus on four aspects: the inhibition of abnormal proliferation of cells, promotion of the apoptosis of tumor cells, antagonism of systemic inflammatory response and the inhibition of angiogenesis. After the analysis of specific signaling pathways, we found that the PI3K-Akt signaling pathway and NF-kappa B signaling pathway are two major pathways of the anti-cancer effect of Wumei Pill on pancreatic neoplasms. We have reasons to believe, the potential mechanism of its anti-cancer effect on pancreatic neoplasms is direct or indirect synergy of multi-target and multi-pathway efforts. However, the further experimental validation is essential to reveal anti-pancreatic cancer effect of the Wumei Pill.

## Additional file

Additional file 1: **Table S1.** Active ingredients and ADME parameters of Wumei Pill (DOCX 890 kb)

## Data Availability

All our main data used to support the findings of this study have been deposited in the figshare repository (10.6084/m9.figshare.7392674.v1). The datasets supporting the conclusions of this article are available in public database from TCMSP, TCM Database@Taiwan, TCMID, SEA, Swiss Target Prediction, SuperPred, CTD, TTD, PharmGKB.

## Abbreviations

ACS: American Cancer Society
DG: Radix Angelicae Sinensis
DL: Drug-likeness
FDR: False discovery rate
FZ: Radix Aconiti Lateralis Praeparata
GJ: Rhizoma Zingiberis
GZ: Ramulus Cinnamomi
HB: Cortex Phellodendri
HJ: Pericarpium Zanthoxyli
HL: Rhizoma Coptidis
OB: Oral Bioavailability
PPI: Protein-protein interaction
RS: Radix Ginseng
TCM: Traditional Chinese Medicine
TS: Tanimoto Similarity
WM: Fructus Mume
WMP: Wumei Pill
XX: Rhizoma Radix Asari

## References

[1] Kamisawa T, Wood LD, Itoi T (2016). Pancreatic cancer. Lancet.

[2] Rahib L, Smith BD, Aizenberg R (2014). Projecting cancer incidence and deaths to 2030: the unexpected burden of thyroid, liver, and pancreas cancers in the United States. Cancer Res.

[3] Smith RA, Brooks D, Cokkinides V (2013). Cancer screening in the United States, 2013: a review of current American Cancer Society guidelines, current issues in cancer screening, and new guidance on cervical cancer screening and lung cancer screening. CA Cancer J Clin.

[4] Sonja G, Tibor S, Christian MZB (2010). Preoperative/neoadjuvant therapy in pancreatic Cancer: a systematic review and meta-analysis of response and resection percentages. PLoS Med.

[5] Bailey P, Chang DK, Nones K (2016). Genomic analyses identify molecular subtypes of pancreatic cancer. Nature.

[6] Liang C, Qin Y, Zhang B (2016). Energy sources identify metabolic phenotypes in pancreatic cancer. Acta Biochim Biophys Sin.

[7] Guohua Y, Wubin W, Xu W (2018). Network pharmacology-based strategy to investigate pharmacological mechanisms of Zuojinwan for treatment of gastritis. BMC Complement Altern Med.

[8] Li M, Wang MM, Guo XW (2018). Different survival benefits of Chinese medicine for pancreatic cancer: how to choose?. Chin J Integr Med.

[9] Kuo YT, Liao HH, Chiang JH (2018). Complementary Chinese herbal medicine therapy improves survival of patients with pancreatic Cancer in Taiwan: a Nationwide population-based cohort study. Integr Cancer Ther.

[10] Mei T, Wang X, Wang A (2015). Effect of Jiaweiwumei decoction on regulatory T cells and interleukin-10 in a rat model of ulcerative colitis. J Tradit Chin Med.

[11] Ma Nai-Xia, Sun Wei, Wu Jian, Liu Shen-Lin, Weng Lei, Liu Yang-Qing, Pu Wen-Xiu, Wu Ting-Ting, Ding Xue-Lian, Huang Nan-Guang, Zheng Pei-Qiu, Zou Xi (2017). Compound Wumei Powder Inhibits the Invasion and Metastasis of Gastric Cancer via Cox-2/PGE2-PI3K/AKT/GSK3β/β-Catenin Signaling Pathway. Evidence-Based Complementary and Alternative Medicine.

[12] Yang Xueping, Li Lingli, Fang Ke, Dong Ruolan, Li Jingbin, Zhao Yan, Dong Hui, Yi Ping, Huang Zhaoyi, Chen Guang, Lu Fuer (2017). Wu-Mei-Wan Reduces Insulin Resistance via Inhibition of NLRP3 Inflammasome Activation in HepG2 Cells. Evidence-Based Complementary and Alternative Medicine.

[13] Jinchang H, Lin X (2012). Clinical observation on 21 cases of pancreatic cancer treated with modified Wumei pills. Chin J Clin.

[14] Hopkins AL (2008). Network pharmacology: the next paradigm in drug discovery. Nat Chem Biol.

[15] Zhang B, Wang X, Li S (2013). An integrative platform of TCM network pharmacology and its application on a herbal formula, Qing-Luo-Yin. Evid Based Complement Alternat Med.

[16] Zhang Y, Bai M, Zhang B (2015). Uncovering pharmacological mechanisms of Wu-tou decoction acting on rheumatoid arthritis through systems approaches: drug-target prediction, network analysis and experimental validation. Sci Rep.

[17] Li H, Zhao L, Zhang B (2014). A network pharmacology approach to determine active compounds and action mechanisms of ge-gen-qin-lian decoction for treatment of type 2 diabetes. Evid Based Complement Alternat Med.

[18] Ru J, Li P, Wang J (2014). TCMSP: a database of systems pharmacology for drug discovery from herbal medicines. J Cheminf.

[19] Chen YC (2011). TCM database@Taiwan: the World's largest traditional Chinese medicine database for drug screening, in silico. PLoS One.

[20] Xue R, Fang Z, Zhang M (2013). TCMID: traditional Chinese medicine integrative database for herb molecular mechanism analysis. Nucleic Acids Res.

[21] Xu X, Zhang W, Huang C (2012). A novel chemometric method for the prediction of human oral bioavailability. Int J Mol Sci.

[22] Willett P, Barnard JM, Downs GM (1998). Chemical similarity searching. J Chem Inf Comput Sci.

[23] Tao W, Xu X, Wang X (2013). Network pharmacology-based prediction of the active ingredients and potential targets of Chinese herbal Radix Curcumae formula for application to cardiovascular disease. J Ethnopharmacol.

[24] Pang KS (2003). Modeling of intestinal drug absorption: roles of transporters and metabolic enzymes (for the gillette review series). Drug Metab Dispos.

[25] Guxiang H, Changhui Z, Wenna Z (2009). QSPR study on the permeability of drugs across Caco-2 monolayer. J Zheijang Univ.

[26] Keiser MJ, Roth BL, Armbruster BN (2007). Relating protein pharmacology by ligand chemistry. Nat Biotechnol.

[27] Gfeller D, Michielin O, Zoete V (2013). Shaping the interaction landscape of bioactive molecules. Bioinformatics.

[28] Nickel J, Gohlke BO, Erehman J (2014). SuperPred: update on drug classification and target prediction. Nucleic Acids Res.

[29] Grondin CJ, Davis AP, Wiegers TC (2018). Accessing an expanded exposure science module at the comparative Toxicogenomics database. Environ Health Perspect.

[30] Li YH, Yu CY, Li XX (2018). Therapeutic target database update 2018: enriched resource for facilitating bench-to-clinic research of targeted therapeutics. Nucleic Acids Res.

[31] Whirl-Carrillo M, Mcdonagh EM, Hebert JM (2012). Pharmacogenomics knowledge for personalized medicine. Clin Pharmacol Ther.

[32] Szklarczyk Damian, Morris John H, Cook Helen, Kuhn Michael, Wyder Stefan, Simonovic Milan, Santos Alberto, Doncheva Nadezhda T, Roth Alexander, Bork Peer, Jensen Lars J., von Mering Christian (2016). The STRING database in 2017: quality-controlled protein–protein association networks, made broadly accessible. Nucleic Acids Research.

[33] Shannon P, Markiel A, Ozier O (2003). Cytoscape: a software environment for integrated models of biomolecular interaction networks. Genome Res.

[34] Missiuro PV, Liu K, Zou L (2009). Information flow analysis of Interactome networks. PLoS Comput Biol.

[35] Raman K, Damaraju N, Joshi GK (2014). The organisational structure of protein networks: revisiting the centrality-lethality hypothesis. Syst Synth Biol.

[36] Tang Y, Li M, Wang J (2015). CytoNCA: a cytoscape plugin for centrality analysis and evaluation of protein interaction networks. Biosystems.

[37] Huang DW, Sherman BT, Lempicki RA (2008). Systematic and integrative analysis of large gene lists using DAVID bioinformatics resources. Nat Protoc.

[38] Chen L, Zhang YH, Wang S (2017). Prediction and analysis of essential genes using the enrichments of gene ontology and KEGG pathways. PLoS One.

[39] Cao ZQ, Wang XX, Lu L (2019). β-Sitosterol and gemcitabine exhibit synergistic anti-pancreatic Cancer activity by modulating apoptosis and inhibiting epithelial–mesenchymal transition by deactivating Akt/GSK-3β signaling. Front Pharmacol.

[40] Lee J, Kim JH (2016). Kaempferol inhibits pancreatic Cancer cell growth and migration through the blockade of EGFR-related pathway in vitro. PLoS One.

[41] Daisuke Hirayama M, Takahiro Fujimori M, Kazuhiro Satonaka M (1992). Immunohisto-chemical study of epidermal growth factor and transforming growth factor-β in the penetrating type of early gastric cancer. Hum Pathol.

[42] Rimawi MF, Shetty PB, Weiss HL (2010). Epidermal growth factor receptor expression in breast cancer association with biologic phenotype and clinical outcomes. Cancer.

[43] Engelman JA, Ji L, Cantley LC (2006). The evolution of phosphatidylinositol 3-kinases as regulators of growth and metabolism. Nat Rev Genet.

[44] Muller PA, Vousden KH (2013). p53 mutations in cancer. Nat Cell Biol.

[45] Lee Jungwhoi, Han Song-I, Yun Jeong-Hun, Kim Jae Hoon (2015). Quercetin 3-O-glucoside suppresses epidermal growth factor–induced migration by inhibiting EGFR signaling in pancreatic cancer cells. Tumor Biology.

[46] Fesik SW (2005). Promoting apoptosis as a strategy for cancer drug discovery. Nat Rev Cancer.

[47] Zhao A, Zeng Q, Xie X (2012). MicroRNA-125b induces cancer cell apoptosis through suppression of Bcl-2 expression. J Genet Genomics.

[48] Duronio V (2008). The life of a cell: apoptosis regulation by the PI3K/PKB pathway. Biochem J.

[49] Wang W, Zeng C, Feng Y (2018). The size-dependent effects of silica nanoparticles on endothelial cell apoptosis through activating the p53-caspase pathway. Environ Pollut.

[50] Murthy KNC, Jayaprakasha GK, Patil BS (2011). Apoptosis mediated cytotoxicity of citrus obacunone in human pancreatic cancer cells. Toxicol in Vitro.

[51] Mantovani A, Allavena P, Sica A (2008). Cancer-related inflammation. Nature.

[52] Sethi Gautam (2008). TNF: A master switch for inflammation to cancer. Frontiers in Bioscience.

[53] Hoesel B, Schmid JA (2013). The complexity of NF-κB signaling in inflammation and cancer. Mol Cancer.

[54] Granado-Serrano AB, Martín MA, Bravo L (2010). Quercetin modulates NF-κ B and AP-1/JNK pathways to induce cell death in human hepatoma cells. Nutr Cancer.

[55] Sabino MC, Ghilardi JR, Feia KJ (2002). The involvement of prostaglandins in tumorigenesis, tumor-induced osteolysis and bone cancer pain. J Musculoskelet Neuronal Interact.

[56] Khorana AA, Ahrendt SA, Ryan CK (2007). Tissue factor expression, angiogenesis, and thrombosis in pancreatic Cancer. Clin Cancer Res.

[57] Liao D, Johnson RS (2007). Hypoxia: a key regulator of angiogenesis in cancer. Cancer Metastasis Rev.

[58] Kim D, Sung B, Kim JA (2017). HS-1793, a resveratrol analogue, downregulates the expression of hypoxia-induced HIF-1 and VEGF and inhibits tumor growth of human breast cancer cells in a nude mouse xenograft model. Int J Oncol.

