# Supplementary material for: Utilising network pharmacology to explore the underlying mechanism of Wumei Pill in treating pancreatic neoplasms
Source: BMC Complement Altern Med. 2019 Jul 4;19:158. doi: 10.1186/s12906-019-2580-y (PMC6611005; doi:10.1186/s12906-019-2580-y)
Supplement: Supplementary file 1 — Table S1. Active ingredients and ADME parameters of Wumei Pill (DOCX 890 kb) [file 12906_2019_2580_MOESM1_ESM.docx]

| **MOL ID** | **Structure** | **Name** | **OB** | **DL** | **Caco-2** | **Herbs** |
| --- | --- | --- | --- | --- | --- | --- |
| MOL000073 | 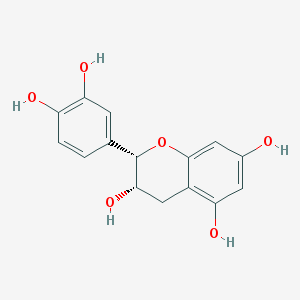 | ent-Epicatechin | 48.96 | 0.24 | 0.02 | GZ |
| MOL000098 | 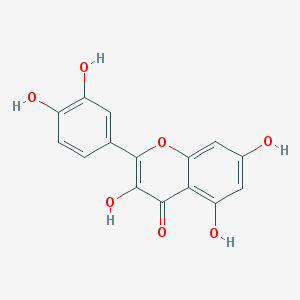 | quercetin | 46.43 | 0.28 | 0.05 | WM/HL/HB |
| MOL000358 | 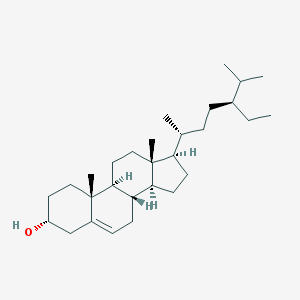 | beta-sitosterol | 36.91 | 0.75 | 1.32 | WM/GJ/DG/HB/GZ/RS |
| MOL000359 | 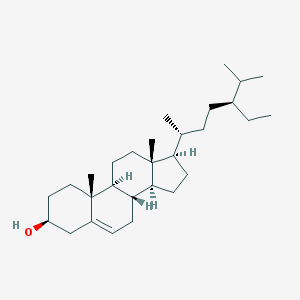 | sitosterol | 36.91 | 0.75 | 1.32 | GJ/FZ/GZ |
| MOL000422 | 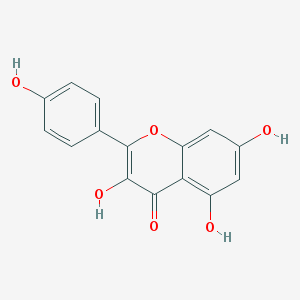 | kaempferol | 41.88 | 0.24 | 0.26 | WM/XX/RS |
| MOL000449 | 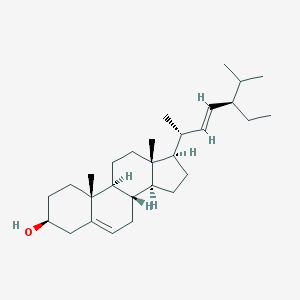 | Stigmasterol | 43.83 | 0.76 | 1.44 | WM/DG/HB/RS |
| MOL000622 | 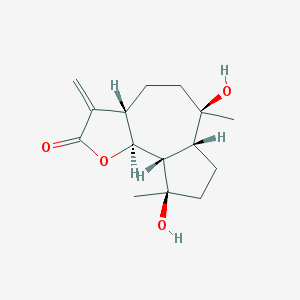 | Magnograndiolide | 63.71 | 0.19 | 0.02 | HL/HB |
| MOL000785 | 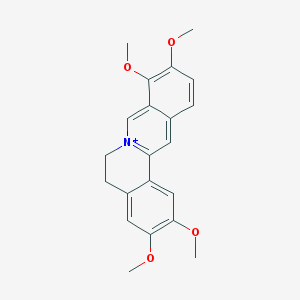 | palmatine | 64.6 | 0.65 | 1.33 | HL/HB |
| MOL000787 | 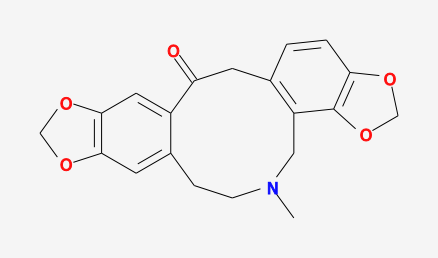 | Fumarine | 59.26 | 0.83 | 0.56 | HB/RS |
| MOL000790 | 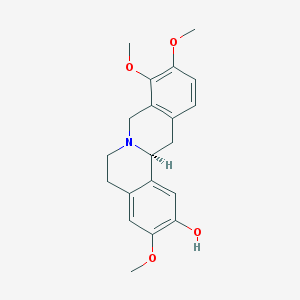 | Isocorypalmine | 35.77 | 0.59 | 0.85 | HB |
| MOL000953 | 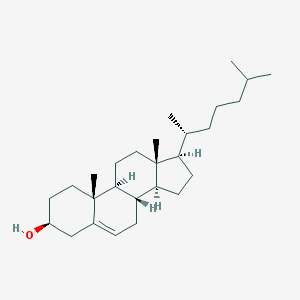 | CLR | 37.87 | 0.68 | 1.43 | WM |
| MOL001040 | 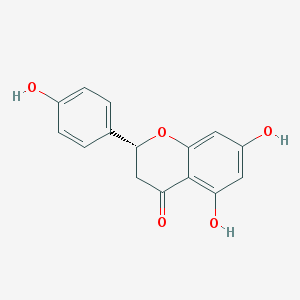 | (2R)-5,7-dihydroxy-2-(4-hydroxyphenyl)chroman-4-one | 42.36 | 0.21 | 0.38 | WM |
| MOL001131 | 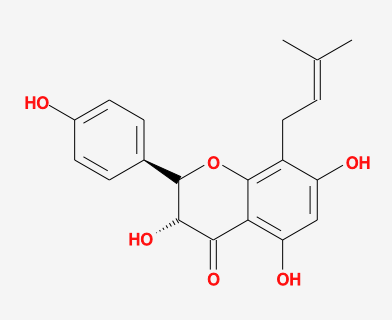 | phellamurin_qt | 56.6 | 0.39 | 0.14 | HB |
| MOL001454 | 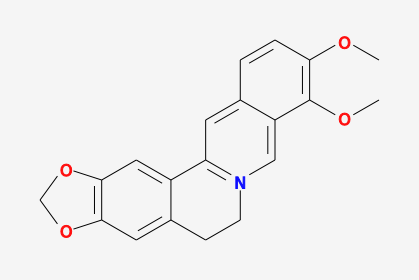 | berberine | 36.86 | 0.78 | 1.24 | HL/HB |
| MOL001455 | 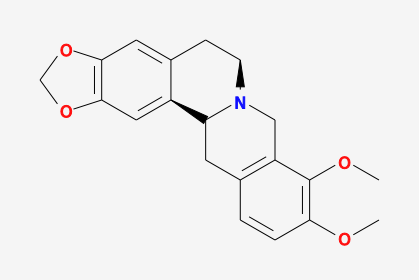 | (S)-Canadine | 53.83 | 0.77 | 1.01 | HB |
| MOL001458 | 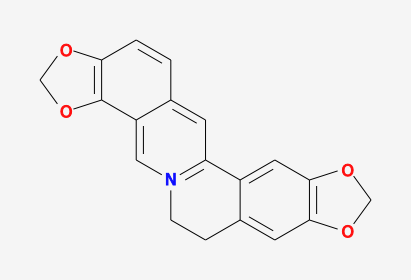 | coptisine | 30.67 | 0.86 | 1.21 | HL/HB |
| MOL001460 | 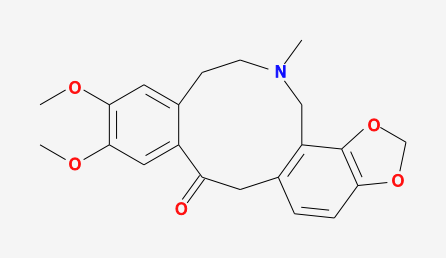 | Cryptopin | 78.74 | 0.72 | 0.79 | XX |
| MOL001558 | 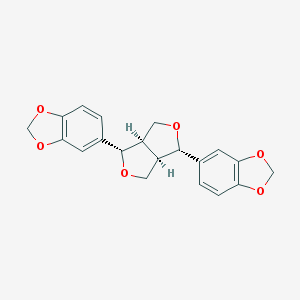 | sesamin | 56.55 | 0.83 | 0.75 | XX |
| MOL001771 | 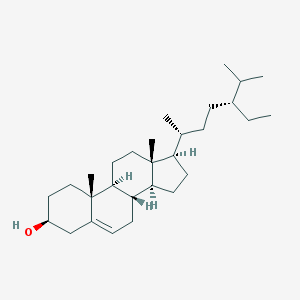 | poriferast-5-en-3beta-ol | 36.91 | 0.75 | 1.45 | HB |
| MOL002211 | 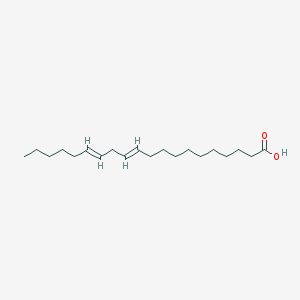 | 11,14-eicosadienoic acid | 39.99 | 0.2 | 1.22 | FZ |
| MOL002331 | 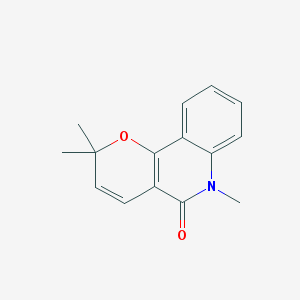 | N-Methylflindersine | 32.36 | 0.18 | 1.35 | HB |
| MOL002392 | 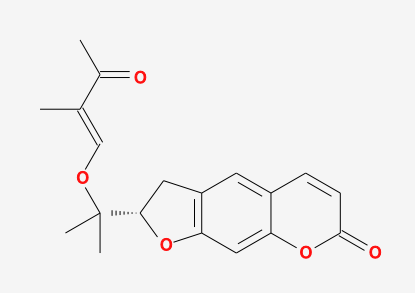 | Deltoin | 46.69 | 0.37 | 0.55 | FZ |
| MOL002395 | 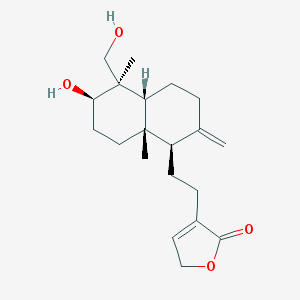 | Deoxyandrographolide | 56.3 | 0.31 | 0.18 | FZ |
| MOL002397 | 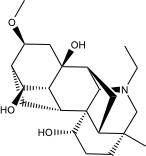 | karakoline | 51.73 | 0.73 | 0.32 | FZ |
| MOL002398 | 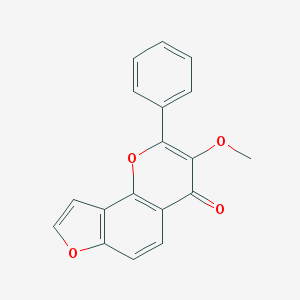 | Karanjin | 69.56 | 0.34 | 1.22 | FZ |
| MOL002401 | 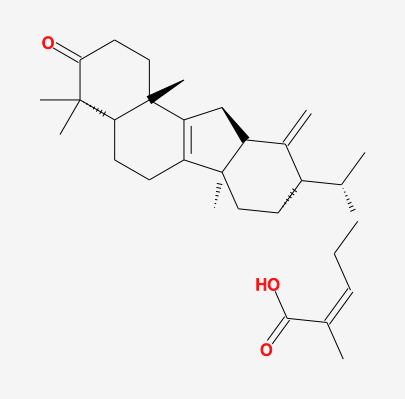 | Neokadsuranic acid B | 43.1 | 0.85 | 0.69 | FZ |
| MOL002410 | 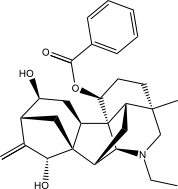 | benzoylnapelline | 34.06 | 0.53 | 0.19 | FZ |
| MOL002419 | 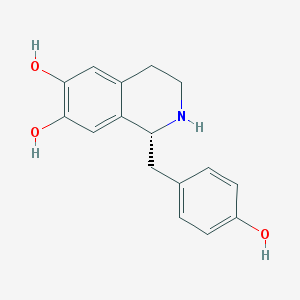 | (R)-Norcoclaurine | 82.54 | 0.21 | 0.63 | FZ |
| MOL002464 | 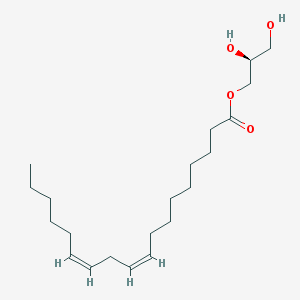 | 1-Monolinolein | 37.18 | 0.3 | 0.32 | GJ |
| MOL002501 | 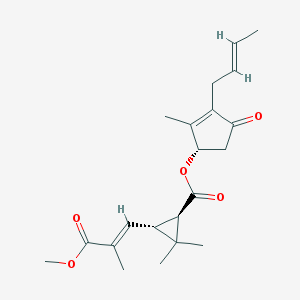 | AC1NSTM8 | 62.52 | 0.31 | 0.37 | XX/GJ |
| MOL002514 | 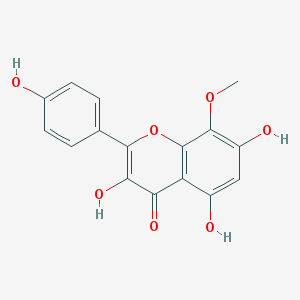 | Sexangularetin | 62.86 | 0.3 | 0.31 | GJ |
| MOL002636 | 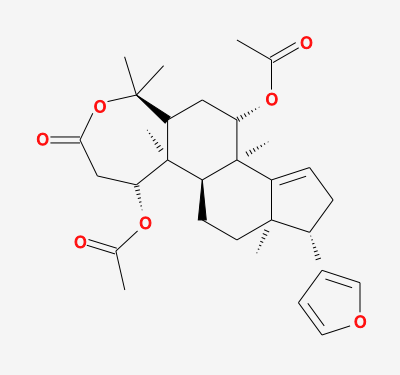 | Kihadalactone A | 34.21 | 0.82 | 0.19 | HB |
| MOL002643 | 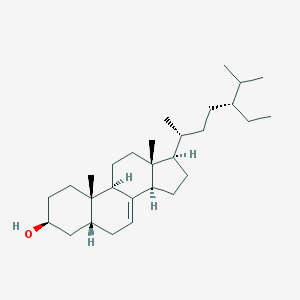 | delta 7-stigmastenol | 37.42 | 0.75 | 1.3 | HB |
| MOL002644 | 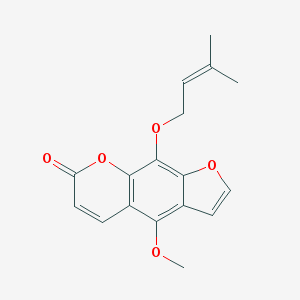 | Phellopterin | 40.19 | 0.28 | 0.98 | HB |
| MOL002651 | 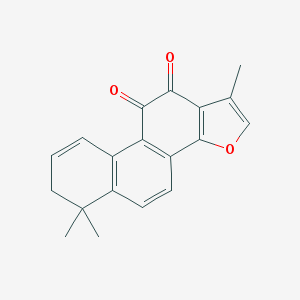 | Dehydrotanshinone II A | 43.76 | 0.4 | 1.02 | HB |
| MOL002652 | 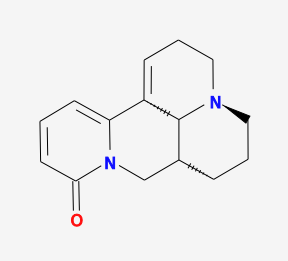 | delta7-Dehydrosophoramine | 54.45 | 0.25 | 0.99 | HB |
| MOL002656 | 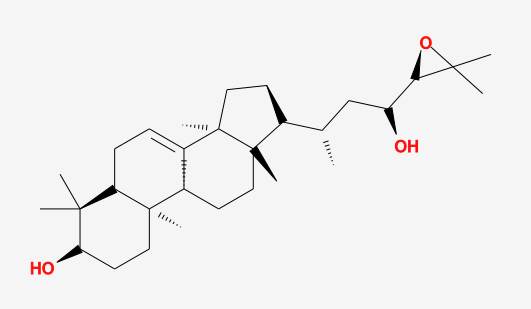 | dihydroniloticin | 36.43 | 0.81 | 0.65 | HB |
| MOL002660 | 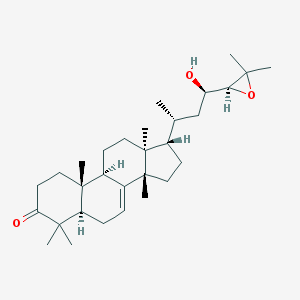 | niloticin | 41.41 | 0.82 | 0.54 | HB |
| MOL002662 | 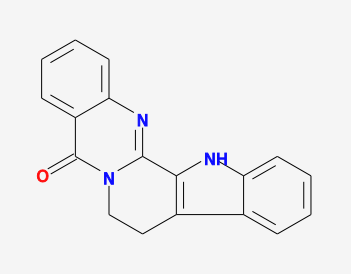 | rutaecarpine | 40.3 | 0.6 | 1.13 | HB |
| MOL002663 | 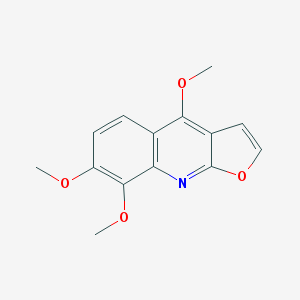 | Skimmianin | 40.14 | 0.2 | 1.26 | HB |
| MOL002666 | 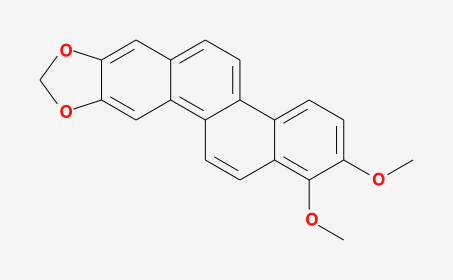 | Chelerythrine | 34.18 | 0.78 | 1.24 | HB |
| MOL002668 | 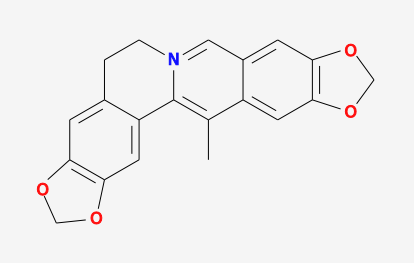 | Worenine | 45.83 | 0.87 | 1.22 | HB/HL |
| MOL002670 | 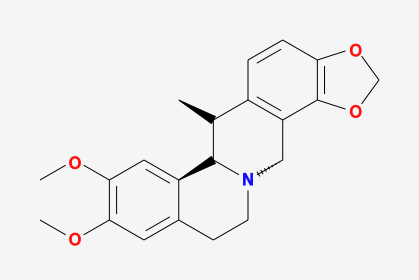 | Cavidine | 35.64 | 0.81 | 1.08 | HB |
| MOL002672 | 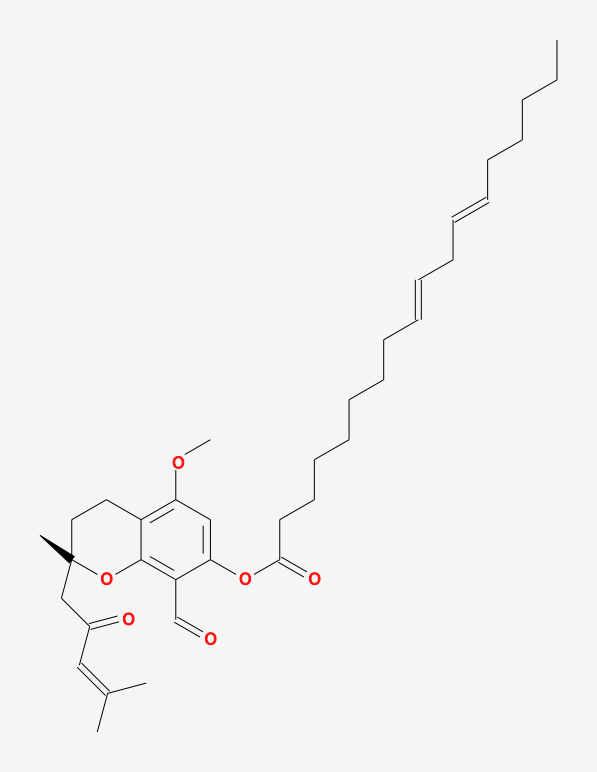 | Hericenone H | 39 | 0.63 | 0.8 | HB |
| MOL002673 | 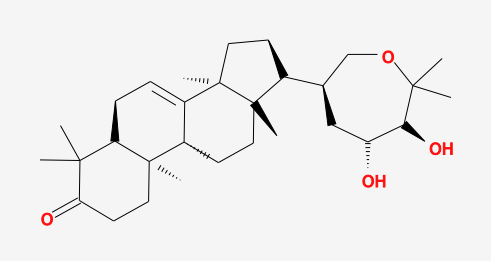 | Hispidone | 36.18 | 0.83 | 0.12 | HB |
| MOL002879 | 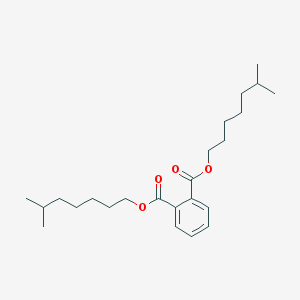 | Diop | 43.59 | 0.39 | 0.79 | RS |
| MOL002894 | 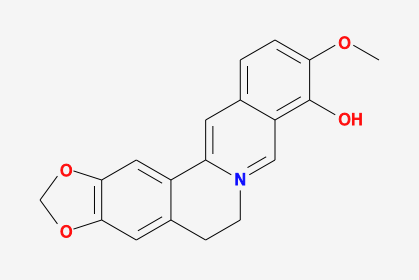 | berberrubine | 35.74 | 0.73 | 1.07 | HB/HL |
| MOL002897 | 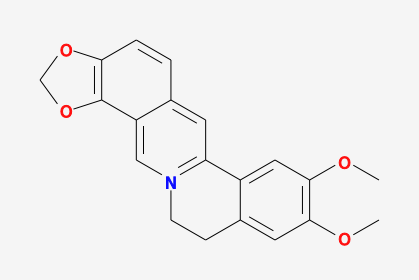 | epiberberine | 43.09 | 0.78 | 1.17 | HL |
| MOL002903 | 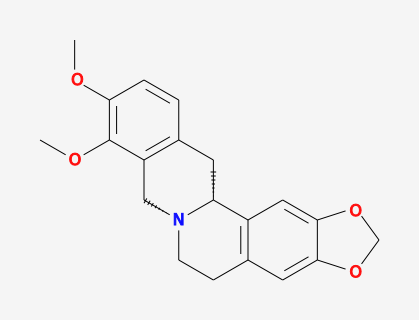 | (R)-Canadine | 55.37 | 0.77 | 1.04 | HL |
| MOL002904 | 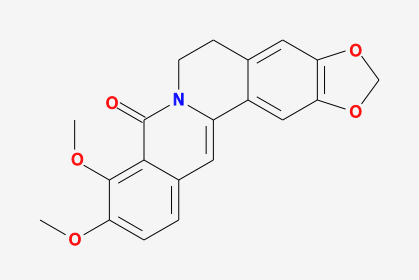 | Berlambine | 36.68 | 0.82 | 0.97 | HL |
| MOL002962 | 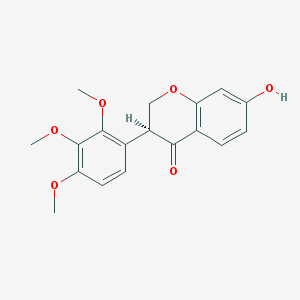 | (3S)-7-hydroxy-3-(2,3,4-trimethoxyphenyl)chroman-4-one | 48.23 | 0.33 | 0.62 | XX |
| MOL003648 | 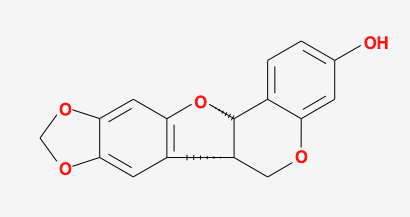 | Inermin | 65.83 | 0.54 | 0.91 | RS |
| MOL004492 | 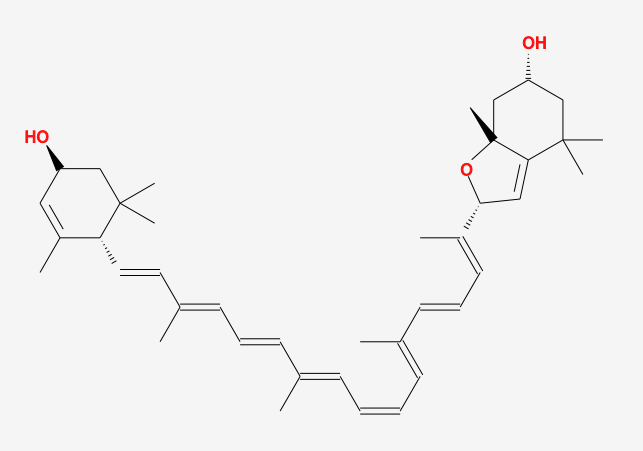 | Chrysanthemaxanthin | 38.72 | 0.58 | 0.51 | RS |
| MOL005043 | 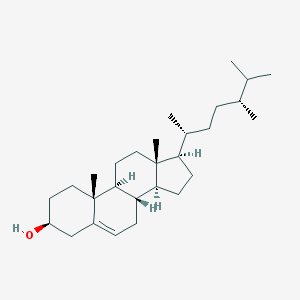 | campest-5-en-3beta-ol | 37.58 | 0.71 | 1.32 | WM |
| MOL005308 | 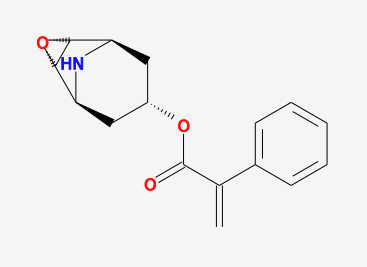 | Aposiopolamine | 66.65 | 0.22 | 0.66 | RS |
| MOL005314 | 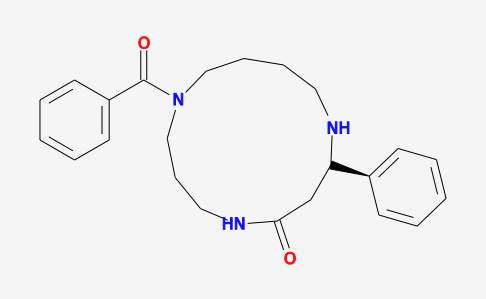 | Celabenzine | 101.88 | 0.49 | 0.77 | RS |
| MOL005317 | 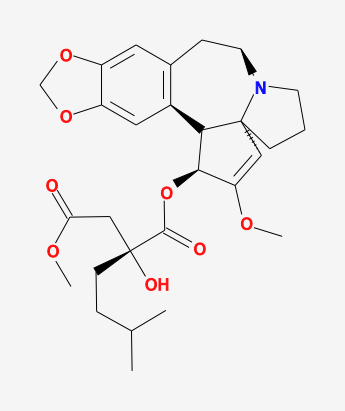 | Deoxyharringtonine | 39.27 | 0.81 | 0.19 | RS |
| MOL005320 | 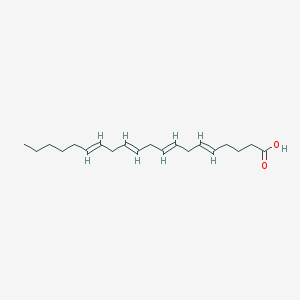 | arachidonate | 45.57 | 0.2 | 1.27 | RS |
| MOL005321 | 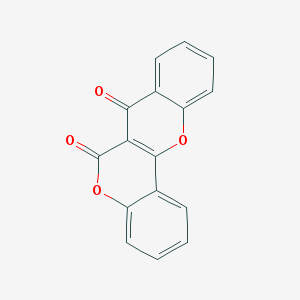 | Frutinone A | 65.9 | 0.34 | 0.89 | RS |
| MOL005348 | 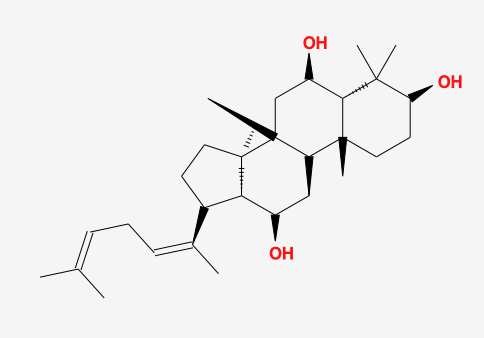 | Ginsenoside-Rh4_qt | 31.11 | 0.78 | 0.5 | RS |
| MOL005356 | 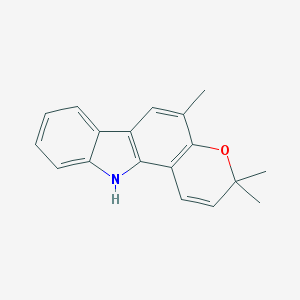 | Girinimbin | 61.22 | 0.31 | 1.72 | RS |
| MOL005357 | 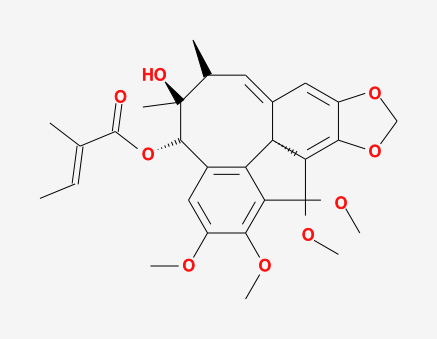 | Gomisin B | 31.99 | 0.83 | 0.6 | RS |
| MOL005360 | 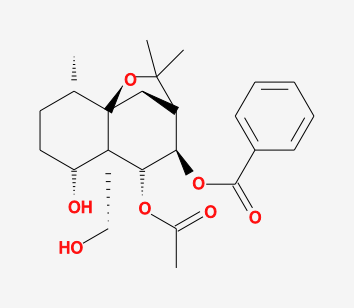 | malkangunin | 57.71 | 0.63 | 0.22 | RS |
| MOL005376 | 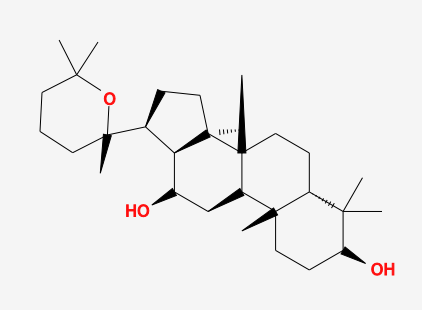 | Panaxadiol | 33.09 | 0.79 | 0.82 | RS |
| MOL005384 | 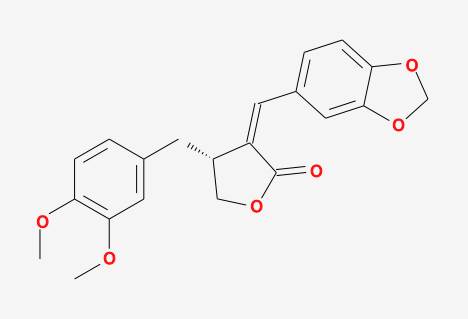 | suchilactone | 57.52 | 0.56 | 0.82 | RS |
| MOL005399 | 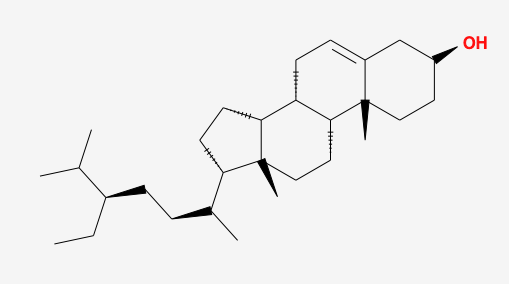 | alexandrin_qt | 36.91 | 0.75 | 1.3 | RS |
| MOL005401 | 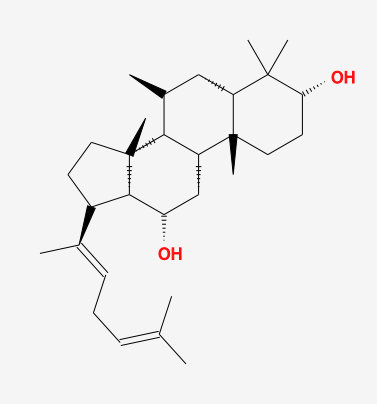 | ginsenoside Rg5_qt | 39.56 | 0.79 | 0.88 | RS |
| MOL005438 | 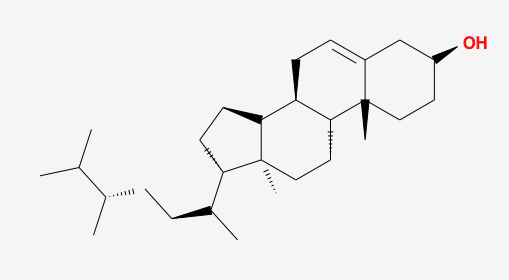 | campesterol | 37.58 | 0.71 | 1.34 | HB |
| MOL006392 | 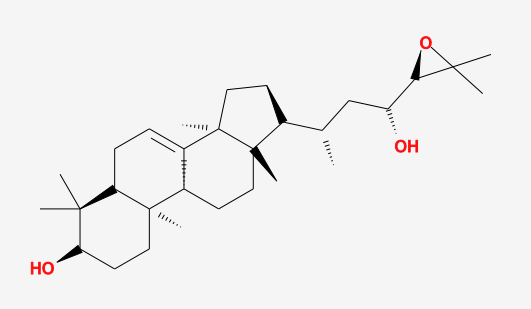 | dihydroniloticin | 36.43 | 0.82 | 0.64 | HB |
| MOL006401 | 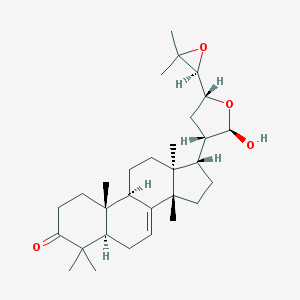 | melianone | 40.53 | 0.78 | 0.4 | HB |
| MOL006413 | 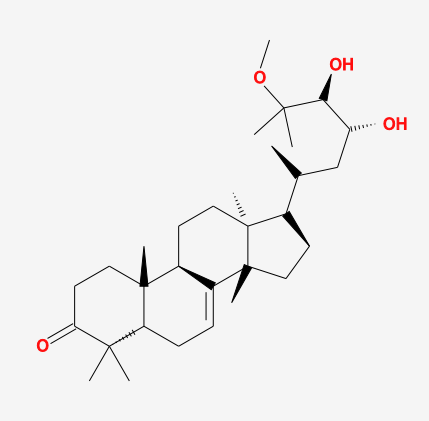 | phellochin | 35.41 | 0.82 | 0.47 | HB |
| MOL006422 | 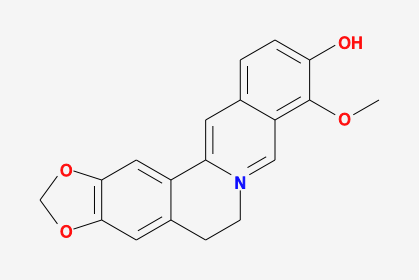 | thalifendine | 44.41 | 0.73 | 1.12 | HB |
| MOL008601 | 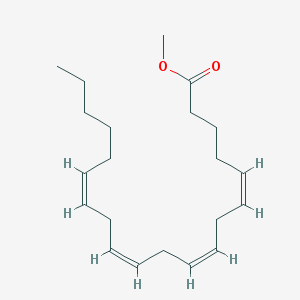 | Methyl arachidonate | 46.9 | 0.23 | 1.48 | WM |
| MOL008647 | 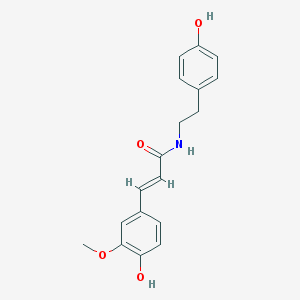 | Moupinamide | 86.71 | 0.26 | 0.55 | HL |
| MOL009849 | 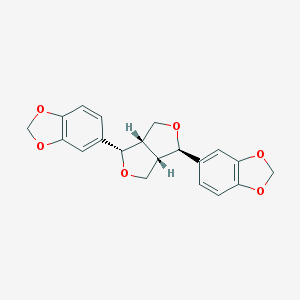 | ZINC05223929 | 31.57 | 0.83 | 0.73 | XX |
| MOL011169 | 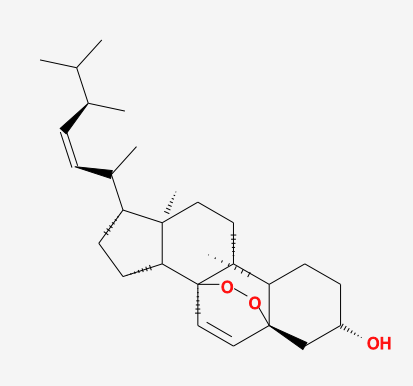 | Peroxyergosterol | 44.39 | 0.82 | 0.86 | GZ |
| MOL012140 | 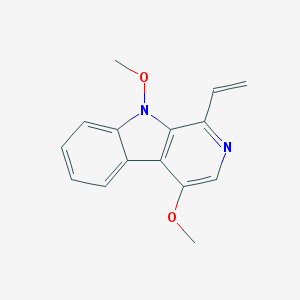 | 4,9-dimethoxy-1-vinyl-$b-carboline | 65.3 | 0.19 | 1.21 | XX |
| MOL012141 | 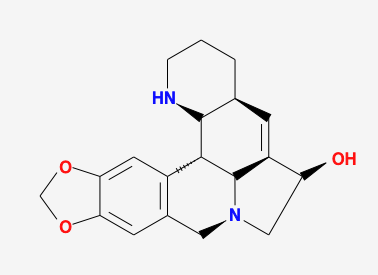 | Caribine | 37.06 | 0.83 | 0.34 | XX |
| MOL013271 | 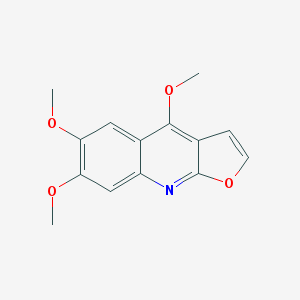 | Kokusaginin | 66.68 | 0.2 | 0.95 | HJ |
| MOL013352 | 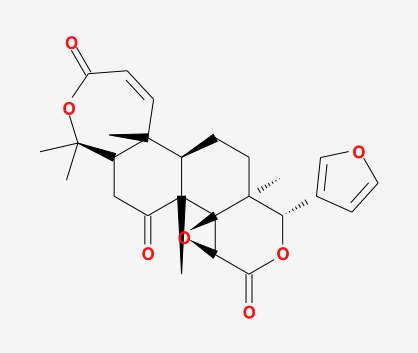 | Obacunone | 43.29 | 0.77 | 0.01 | HB/HL |

**Additional file 1: Table S1. Active ingredients and ADME parameters of Wumei Pill.**
